# Unravelling the Diagnostic Dilemma: A MicroRNA Panel of Circulating MiR-16 and MiR-877 as A Diagnostic Classifier for Distal Bile Duct Tumors

**DOI:** 10.3390/cancers11081181

**Published:** 2019-08-15

**Authors:** Laura L. Meijer, Jisce R. Puik, Tessa Y.S. Le Large, Michal Heger, Frederike Dijk, Niccola Funel, Thomas Wurdinger, Ingrid Garajová, Nicole C.T. van Grieken, Mark A. van de Wiel, Elisa Giovannetti, Geert Kazemier

**Affiliations:** 1 Department of Surgery, Cancer Center Amsterdam, Amsterdam UMC, VU University Amsterdam, 1081 HV, The Netherlands; 2 Department of Medical Oncology, Cancer Center Amsterdam, Amsterdam UMC, VU University Amsterdam, 1081 HV, The Netherlands; 3 Laboratory of Experimental Oncology and Radiobiology, Amsterdam UMC, University of Amsterdam, 1105 AZ, The Netherlands; 4 Department of Experimental Surgery, Amsterdam UMC, University of Amsterdam, 1105 AZ, The Netherlands; 5 Department of Pharmaceutics, Jiaxing University Medical College, Jiaxing 314001, Zhejiang, China; 6 Department of Pharmaceutics, Utrecht Institute of Pharmaceutical Sciences, Utrecht University, 3584 CG, The Netherlands; 7 Department of Pathology, Amsterdam UMC, University of Amsterdam, 1105 AZ, The Netherlands; 8 Cancer Pharmacology Lab, AIRC Start-Up Unit, Fondazione Pisana per la Scienza onlus, 56017 Pisa, Italy; 9 Department of Neurosurgery, Cancer Center Amsterdam, Amsterdam UMC, VU University Amsterdam, 1081 HV, The Netherlands; 10 Medical Oncology Unit, University Hospital of Parma, 43 126 Parma, Italy; 11 Department of Pathology, Cancer Center Amsterdam, Amsterdam UMC, VU University Amsterdam, 1081 HV, The Netherlands; 12 Department of Epidemiology and Biostatistics, Amsterdam Public Health Research Institute, Amsterdam UMC, VU University Amsterdam, 1081 HV, The Netherlands

**Keywords:** biliary tract cancer, distal cholangiocarcinoma, pancreatic cancer, diagnosis, biomarkers

## Abstract

Accurate diagnosis of pancreatic head lesions remains challenging as no minimally invasive biomarkers are available to discriminate distal cholangiocarcinoma (CCA) from pancreatic ductal adenocarcinoma (PDAC). The aim of this study is to identify specific circulating microRNAs (miRNAs) to diagnose distal CCA. In the discovery phase, PCR profiling of 752 miRNAs was performed on fourteen patients with distal CCA and age- and sex-matched healthy controls. Candidate miRNAs were selected for evaluation and validation by RT-qPCR in an independent cohort of distal CCA (*N* = 24), healthy controls (*N* = 32), benign diseases (*N* = 20), and PDAC (*N* = 24). The optimal diagnostic combination of miRNAs was determined by multivariate logistic regression analysis and evaluated by ROC curves with AUC values. The discovery phase revealed 19 significantly dysregulated miRNAs, of which six were validated in the evaluation phase. The validation phase confirmed downregulated miR-16 in patients with distal CCA compared to benign disease or PDAC (*P* = 0.048 and *P* = 0.012), while miR-877 was significantly upregulated (*P* = 0.003 and *P* = 0.006). This two-miRNA panel was validated as a CCA-specific profile, discriminating distal CCA from benign disease (AUC = 0.90) and from PDAC (AUC = 0.88). In conclusion, the present study identified a two-miRNA panel of downregulated miR-16 and upregulated miR-877 with promising capability to diagnose patients with distal CCA.

## 1. Introduction

Adenocarcinomas located in the pancreatic head can be classified as either distal cholangiocarcinoma (CCA) or pancreatic ductal adenocarcinoma (PDAC) [1]. These malignancies show considerable overlap in diagnostic features as patients present with similar symptoms and an indistinguishable mass on imaging modalities [2]. However, distal CCA and PDAC have different outcomes and current treatment regimens differ between these distinct tumor entities [3,4,5]. Distal CCA is classified as CCA based on its anatomic location and treated in analogy with intrahepatic and perihilar CCA [2,6]. Nevertheless, these subtypes have distinct biologic behavior and should thus be considered as individual tumor types [7,8]. Distal CCA is treated with cisplatin combined with gemcitabine, while for PDAC either FOLFIRINOX or gemcitabine plus nanoparticle albumin-bound paclitaxel is used in clinical care [5,9,10]. Diagnostic certainty is needed to optimize correct therapy administration. Although resection of the primary tumor is still the only curative treatment option, neo-adjuvant treatment strategies are gaining momentum and this also urges the need for accurate minimally invasive diagnostic tools.

Currently, the principal diagnostic modalities to diagnose distal CCA preoperatively are imaging and brush cytology or fine needle aspiration (FNA) with cytology of the suspected lesion. Cytological techniques have become routine diagnostics despite the modest sensitivity and the inability to determine tumor origin [11]. In addition, FNA is an invasive procedure which often requires multiple attempts to obtain evaluable specimens [12,13]. Diagnostic certainty is often achieved only after histopathological examination of the resection specimen, although even then distinguishing distal CCA from PDAC can be challenging [14,15,16]. Clinically employed tumor markers in blood, such as carbohydrate antigen 19–9 (CA19–9), show elevated expression levels in patients with distal CCA and PDAC, as well as in patients with benign disease (BD), such as choledocholithiasis and pancreatitis [13,17]. Moreover, obstructive jaundice is a common symptom in patients with pancreatic head lesions, resulting in elevated CA19–9 levels [18]. Consequently, the current diagnostic tools are frequently unable to reliably diagnose and differentiate distal CCA from healthy controls, BD, and PDAC.

In recent years, microRNAs (miRNAs) have shown promising results as minimally invasive diagnostic biomarkers. MiRNAs are small non-coding RNAs that play a central role in post-transcriptional regulation of gene expression and are involved in tumorigenesis [19,20]. Notably, unique miRNA expression profiles are associated with various tumor types, including CCA and PDAC [21,22]. Since miRNAs are highly stable and easily detectable in serum, plasma and other bodily fluids, miRNAs can potentially serve as a novel class of diagnostic biomarkers using easily accessible samples [23,24]. Previous studies evaluated circulating miRNA profiling in patients with PDAC or biliary tract tumors [25,26,27,28]. Nevertheless, no studies investigated the potential of circulating miRNAs to diagnose distal CCA. In this study, we identified plasma miRNAs as minimally invasive diagnostic biomarkers to differentiate distal CCA from healthy individuals, BD, and PDAC.

## 2. Results

### 2.1. Patient Characteristics

The clinical characteristics of study participants in the discovery, evaluation and validation phase are summarized in Table 1. Patients with distal CCA and healthy individuals included in the discovery phase were age- and sex-matched. In the evaluation and validation phase, age of patients with BD was lower compared to distal CCA and PDAC. Patients with PDAC were age-, sex-, and stage-matched with patients with distal CCA to ensure clinical comparability. Interestingly, normal CA19–9 levels were more often found in patients with distal CCA compared to patients with PDAC, although these results were not significant (*P* = 0.056), and median expression levels were comparable between the two groups (*P* = 0.119).

### 2.2. PCR Panel Profiling of MiRNAs in the Discovery Phase

The plasma miRNA PCR panel profiling results of distal CCA compared to healthy individuals are summarized in a heatmap presented in Figure S1A. The initial discovery phase revealed 19 miRNAs to be significantly dysregulated between distal CCA and healthy individuals (Figure S1B). Based on pre-defined selection criteria, 12 miRNAs were selected for further analysis in the evaluation phase (Table S1).

### 2.3. Evaluation of MiRNA Expression in an Independent Cohort Of Distal CCA and Healthy Individuals Reveals Six Dysregulated MiRNAs 

First, stability of candidate reference genes was evaluated in the evaluation and validation phase for normalization of RT-qPCR results [29]. Based on previous studies, four candidate reference miRNAs were included: miR-93, miR-101, miR-39 and miR-1228 [25,30,31,32]. The combination of miR-93 and miR-101 showed the most stable expression across all samples, while miR-1228 expression was undetectable. Thus, the combination of miR-93 and miR-101 was used as reference expression value (Figure S2).

**Figure 1:**
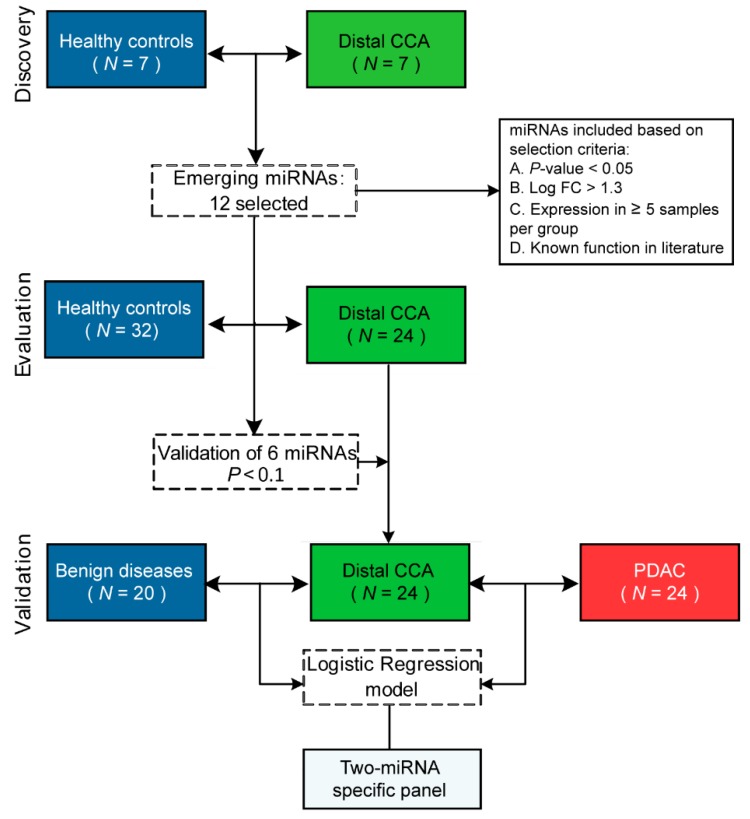
Study design and selection of candidate miRNAs. In the initial discovery phase, PCR panel profiling was performed on seven consecutive patients with distal CCA and seven age- and sex-matched healthy controls. Based on predefined selection criteria, twelve miRNAs were selected for evaluation by RT-qPCR. Of these, six miRNAs were validated to be differentially expressed between distal CCA and healthy controls. These six miRNAs were further validated in the validation phase in patients with distal CCA, benign disease, and PDAC. Distal CCA = distal cholangiocarcinoma, PDAC = pancreatic ductal adenocarcinoma, miRNA = microRNA, Log FC = Log fold change.

**Figure 2:**
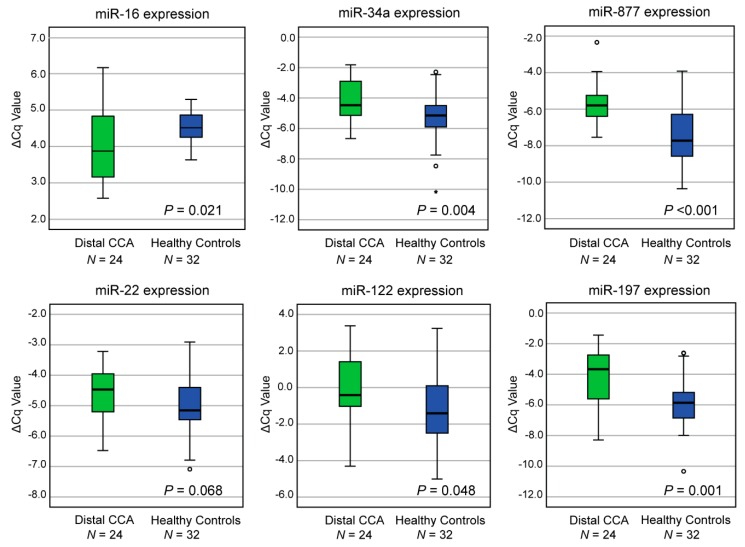
Evaluation of candidate miRNAs in patients with distal CCA compared to healthy individuals. Normalized expression levels (∆Cq values) of each miRNA in the evaluation phase. Expression levels of distal CCA and healthy controls are shown, with horizontal lines representing mean and standard deviation. Distal CCA = distal cholangiocarcinoma.

An independent evaluation cohort of patients with distal CCA (*N* = 24) and healthy controls (*N* = 32) was investigated by RT-qPCR (Figure 1). In this evaluation phase, six miRNAs were confirmed to be dysregulated between distal CCA and healthy controls, while no differences in expression levels were found for the other six miRNAs (Figure 2 and Table S1). In particular, miR-16 was significantly downregulated (*P* = 0.021), while miR-34a (*P* = 0.004), miR-877 (*P* < 0.001), miR-22 (*P* = 0.068), miR-122 (*P* = 0.048), and miR-197 (*P* = 0.001) were upregulated in patients with distal CCA. The ROC-curves with AUC of these individual miRNAs ranged from 0.638 to 0.820 (Figure S3). These six miRNAs were selected for further validation.

### 2.4. Diagnostic Performance of the Optimal MiRNA Panel in the Validation Phase of Distal CCA and BD

**Figure 3:**
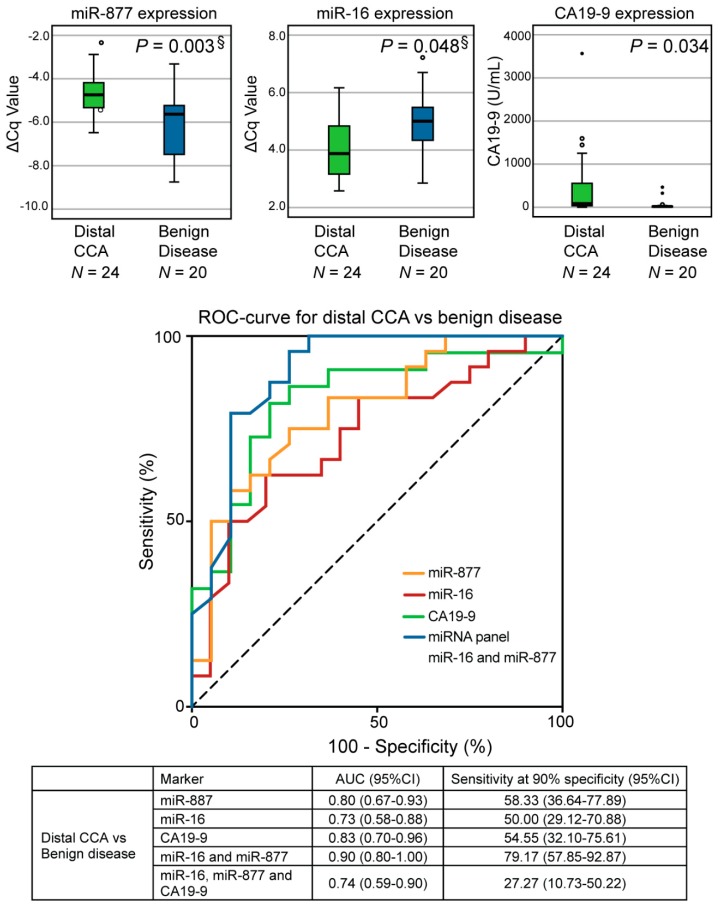
Validation of the selected miRNAs in the validation phase comparing distal CCA and benign disease. Upregulated miR-877, downregulated miR-16 and CA19–9 were significantly differentially expressed in distal CCA compared to benign disease. The two-miRNA panel comprising miR-877 and miR-16 was the most optimal combination (AUC of 0.90) to diagnose distal CCA compared to benign disease. Box plots are displayed for the average ΔCq values, with the horizontal lines representing the mean ± SD. ΔCq = Normalized Cq value, distal CCA = distal cholangiocarcinoma, miRNA = microRNA §Bonferroni-adjusted *P*-values are shown.

The expression levels of candidate miRNAs from plasma samples of patients with distal CCA (*N* = 24) were subsequently compared to patients with BD (*N* = 20). Of the six selected miRNAs for validation, downregulated miR-16 and upregulated miR-877 were differentially expressed in distal CCA versus BD (*P* = 0.048 and *P* = 0.003, respectively, Figure 3). No significantly different expression profiles were found for miR-34a, miR-22, miR-122, and miR-197 when comparing distal CCA and BD (Figure S4). Expression levels of miR-16 were significantly lower in patients with stage III/IV distal CCA (*N* = 5) compared to stage I/II (*N* = 19, *P* = 0.033), while no significant difference was observed for expression levels of miR-877 (Figure S5), although this analysis was limited by the small sample size.

In addition, CA19–9 and bilirubin levels were significantly elevated in patients with distal CCA compared to patients with BD (*P* = 0.034 and *P* < 0.001, respectively; Figure 3 and Figure S6). The most optimal biomarker combination was computed by applying backward logistic regression analyses. Using this strategy, the two-miRNA combination of miR-16 and miR-877 was identified as the most promising combination with an AUC of 0.90 (95%CI: 0.80–1.00, *P* < 0.001, Figure 3). With the threshold for specificity set at 90%, sensitivity was 79% (95%CI: 57.85–92.87). Combining this panel with CA19–9 deteriorated the performance of the panel (AUC = 0.74, 95%CI: 0.59–0.90).

### 2.5. Validation of MiRNA-Based Differentiation between Distal CCA and PDAC

**Figure 4:**
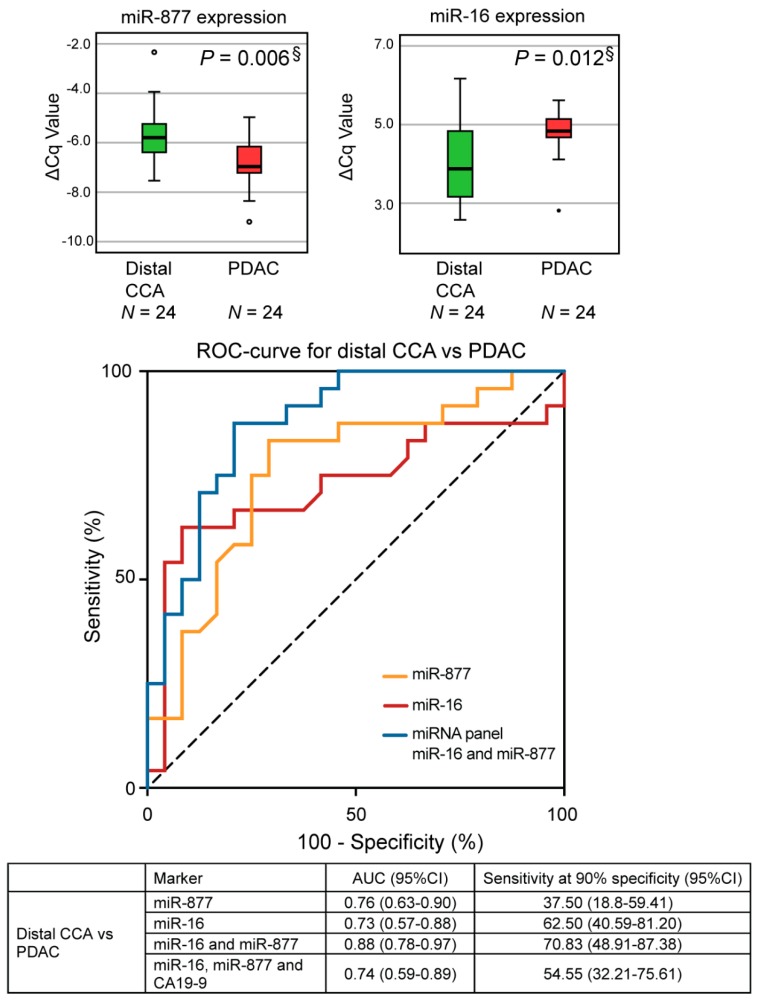
Performance of miRNA-based diagnostics to distinguish distal CCA and PDAC. Expression profiles of miR-877 and miR-16 were significantly different between distal CCA and PDAC (*P* = 0.006 and *P* = 0.012). This two-miRNA panel could accurately distinguish patients with distal CCA from PDAC, with an AUC of 0.88. Box plots are displayed for the average ΔCq values, with the horizontal lines representing the mean ± SD. ΔCq = normalized Cq value, distal CCA = distal cholangiocarcinoma, miRNA = microRNA §Bonferroni-adjusted *P*-values are shown.

To further confirm the diagnostic potential and clinical utility of the two-miRNA panel, miRNA expression profiles of distal CCA (*N* = 24) were compared to age-, sex-, and stage-matched patients with PDAC (*N* = 24). Again, downregulated miR-16 and upregulated miR-877 were significantly different in distal CCA (*P* = 0.012 and *P* = 0.006, respectively, Figure 4). No significant differences in expression profiles were found for miR-34a, miR-22, miR-122, and miR-197 (Figure S4). In addition, there was no significant differential expression of CA19–9 and bilirubin (Table 1).

By applying the same model, the most optimal combination was computed to discriminate distal CCA from PDAC. The combination of miR-16 and miR-877 detected distal CCA with an AUC of 0.88 (95%CI: 0.78–0.98, *P* < 0.001, Figure 4). With a specificity of 90%, our panel resulted in a sensitivity of 71% (95%CI: 48.91–87.38). As expected, combining the two-miRNA panels with CA19–9 did not increase the diagnostic accuracy of this panel (AUC = 0.74, 95%CI: 0.59–0.89). 

### 2.6. Prediction of Target Genes of MiR-16 and MiR-877

By using miRWalk online software, with the most stringent criteria (i.e., 0.95), 22 target genes were predicted for miR-16, while only two target genes were predicted for miR-877, as reported in Table S2. The main targets of miR-16 have been described in several previous studies, showing that they have multiple roles in modulating proliferation of cancer cells [33,34]. Of note, among these targets, FBXW7 has been found to suppress the epithelial–mesenchymal transition, stemness and metastatic potential of cholangiocarcinoma cells [35].

Conversely, only a few data are available for miR-877, including a study showing that the expression of miR-877 was down-regulated in hepatocellular carcinoma tissues or cell lines, while ectopic expression of the target CDK14 reversed the inhibitory effects of miR-877 on proliferation, migration, and invasion of hepatocellular carcinoma cells in vitro [36]. Interestingly, the two targets of miR-877 reported in Table S2 included K-RAS, which plays a major role in both CCA and PDAC carcinogenesis (though *K-RAS* mutations are present in 90% of early stage PDACs, 61% of the ampullary cancers, but only in 15.2% of bile duct cancers [37]). However, SORBS3 recently emerged as a tumor suppressor gene cooperating to inhibit interleukin-6 signaling in hepatocellular carcinoma [38] and is associated with HCC progression [39].

### 2.7. Influence of Bilirubin on the Diagnostic Accuracy of the Two-MiRNA Panel

**Figure 5:**
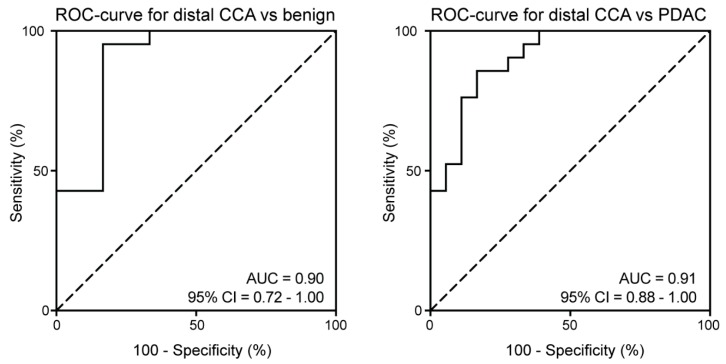
Stratification of patients with high bilirubin levels did not affect the performance of the two-miRNA panel. The two-miRNA panel showed an AUC of 0.90 in patients with elevated bilirubin levels for discriminating patients with distal CCA from benign disease (left) and 0.91 for distal CCA from PDAC (right).

Since bilirubin levels could influence miRNA expression profiles and CA19–9 levels [17,40], the diagnostic power of the two-miRNA panel was also evaluated in patients with high bilirubin levels. High bilirubin levels did not impede the high diagnostic accuracy when comparing distal CCA (*N* = 21) to BD (*N* = 6; AUC=0.90, 95%CI: 0.72–1.00, *P* = 0.004) and to PDAC (*N* = 18; AUC = 0.91, 95%CI: 0.88–1.00, *P* < 0.001, Figure 5).

### 2.8. Expression of MiR-877 and MiR-16 in Plasma Samples of Patients with Distal CCA Compared to Perihilar CCA and Intrahepatic CCA

To examine resemblance of the two-miRNA panel in subtypes of CCA, expression of miR-877 and miR-16 was assessed in plasma samples of patients with perihilar CCA (*N* = 35) and intrahepatic CCA (*N* = 9) compared to distal CCA (*N* = 24, Table S3). Patients with perihilar CCA were more often female (*P* = 0.028) and more patients with stage I/II distal CCA were included (*P* = 0.002). Low levels of bilirubin were more often found in patients with intrahepatic CCA (*P* = 0.005). Expression levels of miR-877 and miR-16 were comparable between subtypes of CCA (Figure S7).

## 3. Discussion

Our research addressed the need for minimally invasive tools to identify distal CCA in current clinical practice. This pioneering study identified a two-miRNA panel of downregulated miR-16 and upregulated miR-877 to accurately diagnose distal CCA. This two-miRNA panel could be particularly useful in patients with suspected distal bile duct tumors to distinguish patients with distal CCA from BD as well as PDAC with high sensitivity and specificity. Moreover, high levels of bilirubin did not impede to performance of the two-miRNA panel. To facilitate translation towards clinical application, cohorts of patients with clinically relevant diagnostic certainty were included.

Novel diagnostic biomarkers are required to ensure appropriate clinical management by distinguishing distal CCA from other lesions in the pancreatic head. Previously, most studies reported molecular profiles of CCA in combined cohorts of extrahepatic and intrahepatic CCA, disregarding the heterogeneity of intrahepatic, perihilar and distal CCA [41,42]. However, appropriate patient stratification is a key determinant in their management and these tumor types should be considered separate entities. Although–omics studies identified common molecular profiles within tumors of the biliary tract, intra- and extra-hepatic CCA show distinct clinical features, etiology, molecular subtypes, and mutational profiles [2,42,43,44]. The comparable expression levels of plasma miR-16 and miR-877 of distal CCA vs perihilar and intrahepatic CCA in the current study underscores this presumed spectrum of CCA, but specific analyses to fully compare plasma miRNA profiles of subtypes of CCA remain to be conducted. Importantly, this similarity in miRNA expression between subtypes of CCA emphasizes the capacity of the two-miRNA panel to distinguish distal CCA from PDAC.

The overrepresentation of intrahepatic CCA in most studies might also explain the discrepancies in miRNAs we found in this study, as we focused specifically on distal CCA. Circulating miR-21 and miR-221 have been described as diagnostic markers for intrahepatic CCA, but the role of these miRNAs was not confirmed as a distal CCA-specific marker in this study [26]. Remarkably, downregulation of miR-16 in CCA was already reported as a diagnostic biomarker, while miR-877 has not been investigated in CCA [45,46]. MiR-16 is characterized as tumor-suppressive miRNA, exerting its function by targeting the Bcl-2-regulated apoptotic pathway [47]. As downregulation of miR-16 has been described in several tumor types, miR-16 could be considered a tumor-associated miRNA [48,49]. Contrary, circulating miR-16 has been reported as a reference gene for normalization due to its stable expression in various tumor types, such as gastric cancer and breast cancer [32,50]. Circulating miRNAs might also originate from blood cells and miR-16 has been correlated to hemolysis [51]. Other miRNAs investigated in this study, including miR-877, miR-34a, and miR-122, did not correlate to blood cells in previous studies. These paradoxical characteristics of miR-16 underscore the importance of standardized sample processing and adequate normalization to minimize the analytical variation, including hemolysis. Together, the combination of miR-16 with miR-877 provides a robust combination, which results in a specific distal CCA diagnostic panel.

Routinely used serum CA19–9 exhibits a wide variation in sensitivity (50–90%) and specificity (54–98%) to diagnose CCA, which precludes CA19–9 as a specific marker to diagnose distal CCA [52]. Strikingly, no research has been performed to investigate the diagnostic accuracy of CA19–9 in patients with distal CCA. In this study, serum CA19–9 expression levels were comparable between patients with distal CCA and PDAC, although more patients with distal CCA displayed normal CA19–9 expression levels. To facilitate the diagnostic process of patients with a suspected malignancy, this two-miRNA panel is superior to CA19–9 with a clinically instrumental AUC (0.88–0.91) for discriminating early-stage distal CCA from BD, as well as from early-stage PDAC. Combining CA19–9 with the two-miRNA panel did not improve the diagnostic power, presumably since expression of CA19–9 was less discriminative between distal CCA and BD, resulting in no additive effect on the diagnostic capacity of the two-miRNA panel.

Additionally, the excellent AUC in the validation phase was established with a clinically relevant benign control group, highlighting the strong discriminative power and clinical utility of the novel two miRNA-based panel. Recent studies investigating the use of a diagnostic blood test, demonstrated comparable sensitivity in diagnosing PDAC [53,54]. However, most studies have focused solely on discrimination of malignant lesions from healthy individuals, neglecting the clinical translation with realistic and relevant control groups, including chronic pancreatitis and choledocholithiasis. This two-miRNA panel is particularly useful in clinical cases which present a diagnostic dilemma, such as comparable clinical symptoms and inconclusive imaging. When clinical suspicion for distal bile duct tumors is present, this panel could help to confirm the specific diagnosis, while its role for diagnostic screening purposes remains to be explored. 

This study is limited by the small sample size of our discovery phase. These results were confirmed by including an independent evaluation and matched validation cohort. Nevertheless, large-scale validation is needed to further verify the diagnostic potential. Additionally, the selected miRNAs included in the discovery phase were deliberately restricted to miRNAs already validated in human plasma samples. This study is the first to apply a broad miRNA discovery approach combined with a confined validation in distal CCA and clinically relevant pancreaticobiliary disease. This focused approach with established protocols ensured accurate detection of the selected miRNAs in liquid biopsies, thereby enhancing practical application. 

To conclude, using a multi-step, statistically robust approach in clinically relevant samples, we discovered a novel two-miRNA panel consisting of miR-16 and miR-877 to detect distal CCA in plasma. This panel is able to discriminate distal CCA from BD and PDAC in patients with clinical suspicion. Our findings are particularly timely since they open up new opportunities to aid in future clinical trials for neo-adjuvant therapies in patients with early-stage tumors, by providing accurate diagnosis at clinical presentation [55].

## 4. Materials and Methods 

### 4.1. Study Design and Patients

The study design and protocol were approved by the local Medical Ethics of the Amsterdam UMC, VU University Amsterdam (VUMC#14438) in accordance with the ethical guidelines of the Declaration of Helsinki. This study was reported in accordance with the Standards for Reporting Diagnostic Accuracy studies (STARD) [56]. Before study participation, written informed consent was obtained from participants.

Blood samples were collected prospectively from all consecutive patients presenting with distal CCA, perihilar CCA, intrahepatic CCA, PDAC, and BD from October 2014 till January 2018. Healthy individuals were enrolled as controls at Amsterdam University Medical Center (UMC), VU University, between 2015 and 2017. Clinicopathological characteristics, including age, sex, tumor stage (AJCC) [57], and tumor marker levels were collected in a prospectively maintained database. 

To identify a diagnostic biomarker profile, this study was designed in three phases (Figure 1). During the discovery phase, PCR panel profiling was performed on seven consecutive patients with distal CCA and seven healthy controls, both age- and sex-matched, enrolled at Amsterdam UMC, VU University. Based on selection criteria, candidate miRNAs were selected for further validation by reverse transcription, real-time quantitative PCR (RT-qPCR). In the evaluation phase, selected miRNAs from the discovery phase were validated in independent cohorts of distal CCA (*N* = 24) and healthy controls (*N* = 32); collected from patients enrolled consecutively at Amsterdam UMC, VU University and Academic Medical Center (AMC), between 2014 and 2018. Dysregulated miRNAs with a *P* < 0.1 were selected for validation in the validation phase to compare the expression profiles of patients with distal CCA to BD (*N* = 20) and age-, sex- and stage-matched PDAC (*N* = 24), collected at Amsterdam UMC, VU University and AMC, and to create a diagnostic panel. Finally, the expression levels of the validated miRNAs were assessed in an additional cohort of patients with perihilar CCA (*N* = 35) and intrahepatic CCA (*N* = 9) compared to distal CCA (*N* = 24) to explore the resemblance between subtypes of CCA.

### 4.2. Sample Collection and MiRNA Expression

Plasma samples were collected at diagnosis and total RNA was extracted using the miRCURY RNA Isolation Kit (Exiqon) according to the manufacturer’s protocol. For the discovery phase, miRNA profiling was performed by PCR panel analysis of 752 miRNAs on the miRNA Human panel I+II (V4, Exiqon) by RT-qPCR. Raw data were normalized to the global mean by subtracting the average of assays detected in all samples from the sample assay Cq (∆Cq) [58]. Following normalization, differential miRNA expression between distal CCA and healthy controls was calculated using the ∆∆Cq method [59]. The PCR panel data have been uploaded to the GEO database (GSE117687).

For the evaluation and validation phase, RNA samples were reverse-transcribed to cDNA and RT-qPCR was performed. A set of four candidate normalizing reference genes was selected for normalization of raw output data in the validation phase, including miR-93, miR-101, miR-39 and miR-1228 [25,29,30,31,32]. NormFinder was used to assess the most stable (combination of) reference gene(s) [60]. MiRNA expression was normalized by the ∆∆Cq method and fold change was expressed as 2∆∆Cq and −2−∆∆Cq for positive and negative ∆Cq, respectively. Detailed methods can be found in the Appendix A—Supplementary Methods and Supplementary Product Information.

### 4.3. Detection of CA19–9 and Bilirubin Levels

Corresponding serum and plasma samples were collected to detect CA19–9 and bilirubin levels for diagnostic purposes at the Clinical Chemistry Laboratory, VUMC (Amsterdam, The Netherlands). The CA19–9 expression levels were determined by the Immunometric assay, Luminescence (Advia Centaur XP, Siemens, USA) and bilirubin levels by the colorimetric diazomethod (Bilirubin Total Gen.3, Roche Diagnostics International, Switzerland). The CA19–9 upper limit of normal (ULN) was set at 37 U/mL and levels were classified as normal, intermediate or high, based on 59 × ULN [61], which resulted in a cut-off of 2183 U/mL. A bilirubin level of ≥20 µmol/L was considered an elevated level of bilirubin.

### 4.4. Statistical Analysis

In the discovery phase, significantly different miRNA expression levels between distal CCA and healthy controls were identified using the unpaired Student’s t-test. Post-hoc analysis was performed with the Benjamini–Hochberg correction method. One-way hierarchical clustering of the miRNAs with a *P*-value of <0.05 was performed to visualize the relative expression level of the miRNAs across distal CCA and healthy controls.

Candidate miRNAs for the evaluation and validation phases were selected based on (i.) *P* < 0.05, (ii.) a log fold change of >1.3, (iii.) expression in ≥5 samples per group, and (iv.) known functions as reported in the literature. In the evaluation phase, differential miRNA expression was first analyzed in an independent cohort of distal CCA and healthy controls using the unpaired Student’s t-test. Expression levels of each miRNA were shown as mean ± standard deviation (SD). Following initial evaluation, miRNAs with *P* < 0.1 were selected and further validated in distal CCA compared to BD and PDAC. In the validation phase, the miRNA expression levels were compared with the unpaired Student’s t-test, followed by post-hoc Bonferroni correction. Univariate analysis was used to select miRNAs, CA19–9, and bilirubin with *P* < 0.05 for subsequent multivariate regression analysis. For construction of the optimal marker panel, multivariate logistic regression analysis followed by backward elimination was performed on the individual miRNAs expression profiles, CA19–9, and bilirubin to select the most optimal combination. Predicted probabilities were calculated for all analyzed samples using the logistic regression model and were used to generate the receiver-operated characteristic (ROC) curve of individual markers, as well as the combined panel. The area under the curve (AUC) with accompanying 95% confidence interval (CI) was calculated to determine its discriminative power. The optimal cut-off point was determined using the Youden Index (J) at high specificity (≥90%) and used to estimate marker sensitivity with 95%CI. In addition, patient groups were stratified to high and normal bilirubin to analyze the diagnostic performance of miRNA panels in patients with high bilirubin levels.

Demographic patient characteristics were compared using the Pearson chi-squared test for nominal variables and Mann–Whitney’s U test or Kruskal–Wallis test for ordinal variables. Depending on the data distribution, the statistical significance of continuous variables was tested with the unpaired Student’s t-test or Mann–Whitney’s U test for comparing two groups, and one-way ANOVA or Kruskal–Wallis analysis for comparing multiple groups. Statistical analysis was performed in SPSS Statistical Software version 24.0 (SPSS, IBM, NY). A *P*-value of ≤0.05 was considered statistically significant.

### 4.5. Prediction of Target Genes of Emerging MiRNAs

Target genes of emerging miRNAs were predicted using the miRWalk version 3.0 (http://mirwalk.umm.uni-heidelberg.de/), which stores predicted data obtained from TargetScan, miRDB and miRTarBase and uses a machine learning algorithm approach, including experimentally verified miRNA-target interactions. Predicted genes that fitted all these databases were considered as target genes.

## 5. Conclusions

This study demonstrates the potential of a two-miRNA panel consisting of miR-16 and miR-877 to detect distal CCA in plasma samples. This two-miRNA panel was identified as a promising diagnostic biomarker combination to discriminate distal CCA from BD and PDAC in patients with clinical suspicion of pancreatic head lesions.

## Figures and Tables

**Table 1:** Clinicopathological Characteristics of the Included Patients.

	Discovery Phase		Evaluation and Validation Phase				
	Healthy control (*N* = 7)	Distal CCA (*N* = 7)	Healthy control (*N* = 32)	Benign disease (*N* = 20)	Distal CCA (*N* = 24)	PDAC (*N* = 24)	*P*-value *
**Age—years**							0.989
Mean (± SD)	68 (± 9)	68 (± 14)	63 (± 9)	60 (± 12)	68 (± 11)	68 (± 10)	
**Sex—No. (%)**							1.000
Male	4 (57)	4 (57)	19 (59)	10 (50)	15 (63)	15 (63)	
Female	3 (43)	3 (43)	13 (41)	10 (50)	9 (37)	9 (37)	
**Stage^+^—No. (%)**							1.000
I	-	0 (0)	-	-	2 (8)	2 (8)	
II	-	6 (86)	-	-	17 (71)	17 (71)	
III	-	1 (14)	-	-	2 (8)	2 (8)	
IV	-	0 (0)	-	-	3 (13)	3 (13)	
**CA19–9—No. (%)**							0.056
Normal §	-	3 (42)	-	15 (75)	6 (25)	1 (4)	
ULN to <59 × ULN	-	2 (29)	-	4 (20)	15 (63)	19 (79)	
High ≥59 × ULN	-	0 (0)	-	0 (0)	1 (4)	4 (17)	
Missing		2 (29)	-	1 (5)	2 (8)	0 (0)	
**CA19–9 (U/mL)**							0.119
Median (± SD)	-	31 (± 337)	-	15 (± 123)	86 (± 844)	461 (± 3114)	
**Bilirubin—No. (%)**							0.267
High	-	6 (86)	0 (0)	6 (30)	21 (87)	18 (75)	
Low	-	1 (14)	31 (97)	13 (65)	3 (13)	6 (25)	
Missing		0 (0)	1 (3)	1 (5)	0 (0)	0 (0)	
**Bilirubin (µmol/L)**							0.908
Median (± SD)	-	52 (± 196)	4 (± 3)	7 (± 26)	104 (± 166)	105 (± 190)	

* Distal CCA compared to PDAC in the evaluation and validation phase. ^+^AJCC Cancer Staging Manual, 7^th^ Edition Distal CCA = distal cholangiocarcinoma, PDAC = pancreatic ductal adenocarcinoma, CA19–9 = carbohydrate antigen 19–9; ULN = Upper Limit of Normal, §The normal range was 0–37 U per milliliter, No. = number of patients.

## Supplementary Materials

The following are available online at https://www.mdpi.com/2072-6694/11/8/1181/s1, Figure S1. A. Heat map diagram showing one-way hierarchical clustering of the differently expressed miRNAs (P < 0.05) emerging from the PCR panel analysis. The heat map reports relative levels of miRNA expression in a green (lower expression) to red (higher expression) scale across all samples. B. Overview of the significantly differently expressed miRNAs in plasma samples of patients with distal CCA compared to healthy controls. Distal CCA = distal cholangicarcinoma, miRNA = microRNA, hsa-miR = human microRNA Figure S2. MiRNA expression of the reference gene combination miR-93 and miR-101 (A) and cel-miR-39 (B) in the evaluation phase and validation phase. Box plots are displayed for the average raw Cq-values, with the horizontal lines representing the mean ± SD. The table (C) displays the stability values of the potential reference miRNAs, as calculated by NormFinder. Figure S3. ROC-curves of the individual microRNAs evaluated in the evaluation phase, comparing distal CCA to healthy controls. ROC-curves with AUC are displayed. Figure S4. Individual miRNA expression profiles of the miRNAs investigated in the validation phase. Expression levels of miR-34a (A), miR-22 (B), miR-122 (C), and miR-197 (D) comparing distal CCA to benign disease and PDAC. No significant differences were found for these miRNAs. Normalized Cq (ΔCq) are shown, box plots are displayed for the average ΔCq-values, with the horizontal lines representing the mean ± SD. Figure S5. Expression of miR-16 (A) and miR-877 (B) in early stage (I/II) and late stage (III/IV) distal cholangiocarcinoma. Expressions of miR-16 was significantly lower in late stage distal cholangiocarcinoma.Figure S6. Expression of bilirubin levels in patients with benign disease versus distal CCA and distal CCA versus PDAC. Figure S7. Expression levels of miR-877 (A) and miR-16 (B) in plasma samples of patients with distal CCA compared to perihilar and intrahepatic CCA. No significant differences were found between the expression levels of the groups. Normalized Cq (ΔCq) are shown, box plots are displayed for the average Cq-values, with the horizontal lines representing the mean ± SD. CCA = cholangiocarcinoma. Table S1—Overview of the 12 miRNAs selected for evaluation and the P-value comparing distal CCA to healthy controls in the evaluation phase. Table S2. Predicted gene targets of miR-16–5p and miR-877–5p. Table S3. Clinicopathological characteristics of the included patients with distal CCA, perihilar CCA, and intrahepatic CCA.

## References

[1] Bledsoe J.R., Shinagare S.A., Deshpande V. (2014). Difficult Diagnostic Problems in Pancreatobiliary Neoplasia. Arch. Pathol. Lab. Med..

[2] Blechacz B., Komuta M., Roskams T., Gores G.J. (2011). Clinical diagnosis and staging of cholangiocarcinoma. Nat. Rev. Gastroenterol. Hepatol..

[3] Ethun C.G., Lopez-Aguiar A.G., Pawlik T.M., Poultsides G., Idrees K., Fields R.C., Weber S.M., Cho C., Martin R.C., Scoggins C.R. (2017). Distal Cholangiocarcinoma and Pancreas Adenocarcinoma: Are They Really the Same Disease? A 13-Institution Study from the US Extrahepatic Biliary Malignancy Consortium and the Central Pancreas Consortium. J. Am. Coll. Surg..

[4] Caparello C., Meijer L.L., Garajova I., Falcone A., Le Large T.Y., Funel N., Kazemier G., Peters G.J., Vasile E., Giovannetti E. (2016). Folfirinox and translational studies: Towards personalized therapy in pancreatic cancer. World J. Gastroenterol..

[5] Valle J.W., Furuse J., Jitlal M., Beare S., Mizuno N., Wasan H., Bridgewater J., Okusaka T. (2014). Cisplatin and gemcitabine for advanced biliary tract cancer: A meta-analysis of two randomised trials. Ann. Oncol..

[6] Valle J., Wasan H., Palmer D.H., Cunningham D., Anthoney A., Maraveyas A., Madhusudan S., Iveson T., Hughes S., Pereira S.P. (2010). Cisplatin plus gemcitabine versus gemcitabine for biliary tract cancer. N. Engl. J. Med..

[7] Rizvi S., Gores G.J. (2017). Emerging molecular therapeutic targets for cholangiocarcinoma. J. Hepatol..

[8] Yachida S., Wood L.D., Suzuki M., Takai E., Totoki Y., Kato M., Luchini C., Arai Y., Nakamura H., Hama N. (2016). Genomic Sequencing Identifies ELF3 as a Driver of Ampullary Carcinoma. Cancer Cell.

[9] Conroy T., Desseigne F., Ychou M., Bouche O., Guimbaud R., Becouarn Y., Adenis A., Raoul J.L., Gourgou-Bourgade S., De La Fouchardiere C. (2011). FOLFIRINOX versus gemcitabine for metastatic pancreatic cancer. N. Engl. J. Med..

[10] Von Hoff D.D., Ervin T., Arena F.P., Chiorean E.G., Infante J., Moore M., Seay T., Tjulandin S.A., Ma W.W., Saleh M.N. (2013). Increased survival in pancreatic cancer with nab-paclitaxel plus gemcitabine. N. Engl. J. Med..

[11] Korc P., Sherman S. (2016). ERCP tissue sampling. Gastrointest Endosc.

[12] Weynand B., Deprez P. (2004). Endoscopic ultrasound guided fine needle aspiration in biliary and pacreatic diseases: Pitfalls and performances. Acta Gastro Enterol. Belg..

[13] Khan S.A., Davidson B.R., Goldin R.D., Heaton N., Karani J., Pereira S.P., Rosenberg W.M., Tait P., Taylor-Robinson S.D., Thillainayagam A.V. (2012). British Society of Gastroenterology, Guidelines for the diagnosis and treatment of cholangiocarcinoma: An update. Gut.

[14] Davidson B.R., Gurusamy K. (2008). Is preoperative histological diagnosis necessary for cholangiocarcinoma?. HPB.

[15] Soer E., Brosens L., Van De Vijver M., Dijk F., Van Velthuysen M.L., Farina-Sarasqueta A., Morreau H., Offerhaus J., Koens L., Verheij J. (2018). Dilemmas for the pathologist in the oncologic assessment of pancreatoduodenectomy specimens: An overview of different grossing approaches and the relevance of the histopathological characteristics in the oncologic assessment of pancreatoduodenectomy specimens. Virchows Arch..

[16] Pomianowska E., Grzyb K., Westgaard A., Clausen O.P., Gladhaug I.P. (2012). Reclassification of tumour origin in resected periampullary adenocarcinomas reveals underestimation of distal bile duct cancer. Eur. J. Surg. Oncol..

[17] Ballehaninna U.K., Chamberlain R.S. (2012). The clinical utility of serum CA 19–9 in the diagnosis, prognosis and management of pancreatic adenocarcinoma: An evidence based appraisal. J. Gastrointest. Oncol..

[18] Marrelli D., Caruso S., Pedrazzani C., Neri A., Fernandes E., Marini M., Pinto E., Roviello F. (2009). CA19–9 serum levels in obstructive jaundice: Clinical value in benign and malignant conditions. Am. J. Surg..

[19] Ha M., Kim V.N. (2014). Regulation of microRNA biogenesis. Nat. Rev. Mol. Cell Biol..

[20] Bartel D.P. (2009). MicroRNAs: Target recognition and regulatory functions. Cell.

[21] Hayes J., Peruzzi P.P., Lawler S. (2014). MicroRNAs in cancer: Biomarkers, functions and therapy. Trends Mol. Med..

[22] Calin G.A., Croce C.M. (2006). MicroRNA signatures in human cancers. Nat. Rev. Cancer.

[23] Verhoeven C.J., Farid W.R., De Jonge J., Metselaar H.J., Kazemier G., Van Der Laan L.J. (2014). Biomarkers to assess graft quality during conventional and machine preservation in liver transplantation. J. Hepatol..

[24] Cortez M.A., Bueso-Ramos C., Ferdin J., Lopez-Berestein G., Sood A.K., Calin G.A. (2011). MicroRNAs in body fluids—The mix of hormones and biomarkers. Nat. Rev. Clin. Oncol..

[25] Pei Z., Liu S.-M., Huang J.-T., Zhang X., Yan D., Xia Q., Ji C., Chen W., Zhang X., Xu J. (2017). Clinically relevant circulating microRNA profiling studies in pancreatic cancer using meta-analysis. Oncotarget.

[26] Correa-Gallego C., Maddalo D., Doussot A., Kemeny N., Kingham T.P., Allen P.J., D’Angelica M.I., DeMatteo R.P., Betel D., Klimstra D. (2016). Circulating Plasma Levels of MicroRNA-21 and MicroRNA-221 Are Potential Diagnostic Markers for Primary Intrahepatic Cholangiocarcinoma. PLoS ONE.

[27] Letelier P., Riquelme I., Hernandez A.H., Guzman N., Farias J.G., Roa J.C. (2016). Circulating MicroRNAs as Biomarkers in Biliary Tract Cancers. Int. J. Mol. Sci..

[28] Zheng B., Jeong S., Zhu Y., Chen L., Xia Q. (2017). miRNA and lncRNA as biomarkers in cholangiocarcinoma(CCA). Oncotarget.

[29] Schwarzenbach H., Da Silva A.M., Calin G., Pantel K. (2015). Data Normalization Strategies for MicroRNA Quantification. Clin. Chem..

[30] Hu J., Wang Z., Liao B.Y., Yu L., Gao X., Lu S., Wang S., Dai Z., Zhang X., Chen Q. (2014). Human miR-1228 as a stable endogenous control for the quantification of circulating microRNAs in cancer patients. Int. J. Cancer.

[31] Niu Y., Wu Y., Huang J., Li W., Kang K., Qu J., Gou D. (2016). Identification of reference genes for circulating microRNA analysis in colorectal cancer. Sci. Rep..

[32] Song J., Bai Z., Han W., Zhang J., Meng H., Bi J., Ma X., Han S., Zhang Z. (2012). Identification of suitable reference genes for qPCR analysis of serum microRNA in gastric cancer patients. Dig. Dis. Sci..

[33] Wang K., Li P., Dong Y., Cai X., Hou D., Guo J., Yin Y., Zhang Y., Li J., Liang H. (2011). A microarray-based approach identifies ADP ribosylation factor-like protein 2 as a target of microRNA-16. J. Biol. Chem..

[34] Yan X., Liang H., Deng T., Zhu K., Zhang S., Wang N., Jiang X., Wang X., Liu R., Zen K. (2013). The identification of novel targets of miR-16 and characterization of their biological functions in cancer cells. Mol. Cancer.

[35] Yang H., Lu X., Liu Z., Chen L., Xu Y., Wang Y., Wei G., Chen Y. (2015). FBXW7 suppresses epithelial-mesenchymal transition, stemness and metastatic potential of cholangiocarcinoma cells. Oncotarget.

[36] Yan T.H., Qiu C., Sun J., Li W.H. (2018). MiR-877–5p suppresses cell growth, migration and invasion by targeting cyclin dependent kinase 14 and predicts prognosis in hepatocellular carcinoma. Eur. Rev. Med. Pharm. Sci..

[37] Schmuck R.B., De Carvalho-Fischer C.V., Neumann C., Pratschke J., Bahra M. (2016). Distal bile duct carcinomas and pancreatic ductal adenocarcinomas: Postulating a common tumor entity. Cancer Med..

[38] Ploeger C., Waldburger N., Fraas A., Goeppert B., Pusch S., Breuhahn K., Wang X.W., Schirmacher P., Roessler S. (2016). Chromosome 8p tumor suppressor genes SH2D4A and SORBS3 cooperate to inhibit interleukin-6 signaling in hepatocellular carcinoma. Hepatology.

[39] Roessler S., Long E.L., Budhu A., Chen Y., Zhao X., Ji J., Walker R., Jia H.L., Ye Q.H., Qin L.X. (2012). Integrative genomic identification of genes on 8p associated with hepatocellular carcinoma progression and patient survival. Gastroenterology.

[40] Yamaura Y., Nakajima M., Takagi S., Fukami T., Tsuneyama K., Yokoi T. (2012). Plasma microRNA profiles in rat models of hepatocellular injury, cholestasis, and steatosis. PLoS ONE.

[41] Puik J.R., Meijer L.L., Le Large T.Y., Prado M.M., Frampton A.E., Kazemier G., Giovannetti E. (2017). miRNA profiling for diagnosis, prognosis and stratification of cancer treatment in cholangiocarcinoma. Pharmacogenomics.

[42] Nakamura H., Arai Y., Totoki Y., Shirota T., Elzawahry A., Kato M., Hama N., Hosoda F., Urushidate T., Ohashi S. (2015). Genomic spectra of biliary tract cancer. Nat. Genet..

[43] Komuta M., Govaere O., Vandecaveye V., Akiba J., Van Steenbergen W., Verslype C., Laleman W., Pirenne J., Aerts R., Yano H. (2012). Histological diversity in cholangiocellular carcinoma reflects the different cholangiocyte phenotypes. Hepatology.

[44] Jusakul A., Cutcutache I., Yong C.H., Lim J.Q., Huang M.N., Padmanabhan N., Nellore V., Kongpetch S., Ng A.W.T., Ng L.M. (2017). Whole-Genome and Epigenomic Landscapes of Etiologically Distinct Subtypes of Cholangiocarcinoma. Cancer Discov..

[45] Han S., Wang D., Tang G., Yang X., Jiao C., Yang R., Zhang Y., Huo L., Shao Z., Lu Z. (2017). Suppression of miR-16 promotes tumor growth and metastasis through reversely regulating YAP1 in human cholangiocarcinoma. Oncotarget.

[46] Kojima M., Sudo H., Kawauchi J., Takizawa S., Kondou S., Nobumasa H., Ochiai A. (2015). MicroRNA markers for the diagnosis of pancreatic and biliary-tract cancers. PLoS ONE.

[47] Aqeilan R.I., Calin G.A., Croce C.M. (2010). miR-15a and miR-16–1 in cancer: Discovery, function and future perspectives. Cell Death Differ..

[48] Bonci D., Coppola V., Musumeci M., Addario A., Giuffrida R., Memeo L., D’Urso L., Pagliuca A., Biffoni M., Labbaye C. (2008). The miR-15a-miR-16–1 cluster controls prostate cancer by targeting multiple oncogenic activities. Nat. Med..

[49] Calin G.A., Dumitru C.D., Shimizu M., Bichi R., Zupo S., Noch E., Aldler H., Rattan S., Keating M., Rai K. (2002). Frequent deletions and down-regulation of micro- RNA genes miR15 and miR16 at 13q14 in chronic lymphocytic leukemia. Proc. Natl. Acad. Sci. USA.

[50] Heneghan H.M., Miller N., Lowery A.J., Sweeney K.J., Newell J., Kerin M.J. (2010). Circulating microRNAs as novel minimally invasive biomarkers for breast cancer. Ann. Surg..

[51] Pritchard C.C., Kroh E., Wood B., Arroyo J.D., Dougherty K.J., Miyaji M.M., Tait J.F., Tewari M. (2012). Blood cell origin of circulating microRNAs: A cautionary note for cancer biomarker studies. Cancer Prev. Res..

[52] Sandanayake N.S., Sinclair J., Andreola F., Chapman M.H., Xue A., Webster G.J., Clarkson A., Gill A., Norton I.D., Smith R.C. (2011). A combination of serum leucine-rich alpha-2-glycoprotein 1, CA19–9 and interleukin-6 differentiate biliary tract cancer from benign biliary strictures. Br. J. Cancer.

[53] Cohen J.D., Li L., Wang Y., Thoburn C., Afsari B., Danilova L., Douville C., Javed A.A., Wong F., Mattox A. (2018). Detection and localization of surgically resectable cancers with a multi-analyte blood test. Science.

[54] Cohen J.D., Javed A.A., Thoburn C., Wong F., Tie J., Gibbs P., Schmidt C.M., Yip-Schneider M.T., Allen P.J., Schattner M. (2017). Combined circulating tumor DNA and protein biomarker-based liquid biopsy for the earlier detection of pancreatic cancers. Proc. Natl. Acad. Sci. USA.

[55] Versteijne E., Van Eijck C.H., Punt C.J., Suker M., Zwinderman A.H., Dohmen M.A., Groothuis K.B., Busch O.R., Besselink M.G., De Hingh I.H. (2016). Preoperative radiochemotherapy versus immediate surgery for resectable and borderline resectable pancreatic cancer (PREOPANC trial): Study protocol for a multicentre randomized controlled trial. Trials.

[56] Bossuyt P.M., Reitsma J.B., Bruns D.E., Gatstonis C.A., Glasziou P.P., Irwig L., Lijmer J.G., Moher D., Rennie D., De Vet H.C.W. (2015). STARD 2015: An Updated List of Essential Items for Reporting Diagnostic Accuracy Studies. Clin. Chem..

[57] Edge S.B., Compton C.C. (2010). The American Joint Committee on Cancer: The 7th Edition of the AJCC Cancer Staging Manual and the Future of TNM. Ann. Surg. Oncol..

[58] Mestdagh P., Van Vlierberghe P., De Weer A., Muth D., Westermann F., Speleman F., Vandesompele J. (2009). A novel and universal method for microRNA RT-qPCR data normalization. Genome Biol..

[59] Livak K.J., Schmittgen T.D. (2001). Analysis of relative gene expression data using real-time quantitative PCR and the 2 (-Delta Delta C(T)) Method. Methods.

[60] Andersen C.L., Jensen J.L., Orntoft T.F. (2004). Normalization of real-time quantitative reverse transcription-PCR data: A model-based variance estimation approach to identify genes suited for normalization, applied to bladder and colon cancer data sets. Cancer Res..

[61] Hess V., Glimelius B., Grawe P., Dietrich D., Bodoky G., Ruhstaller T., Bajetta E., Saletti P., Figer A., Scheithauer W. (2008). CA 19–9 tumour-marker response to chemotherapy in patients with advanced pancreatic cancer enrolled in a randomised controlled trial. Lancet Oncol..

