# Supplementary material for: Unravelling the Diagnostic Dilemma: A MicroRNA Panel of Circulating MiR-16 and MiR-877 as A Diagnostic Classifier for Distal Bile Duct Tumors
Source: Cancers (Basel). 2019 Aug 15;11(8):1181. doi: 10.3390/cancers11081181 (PMC6721566; doi:10.3390/cancers11081181)
Supplement: Supplementary file 1 [file cancers-11-01181-s001.zip › cancers-563234-supplyment update/cancers-563234-Supplementary Materials.docx]

Supplementary Materials

Unravelling the diagnostic dilemma: A miRNA panel of circulating miR-16 and miR-877 as diagnostic classifier for distal bile duct tumors

Laura L. Meijer; Jisce R. Puik; Tessa Y.S. Le Large; Michal Heger; Frederike Dijk; Niccola Funel; Thomas Wurdinger; Ingrid Garajová; Nicole C.T. van Grieken; Mark A. van de Wiel; Elisa Giovannetti; and Geert Kazemier


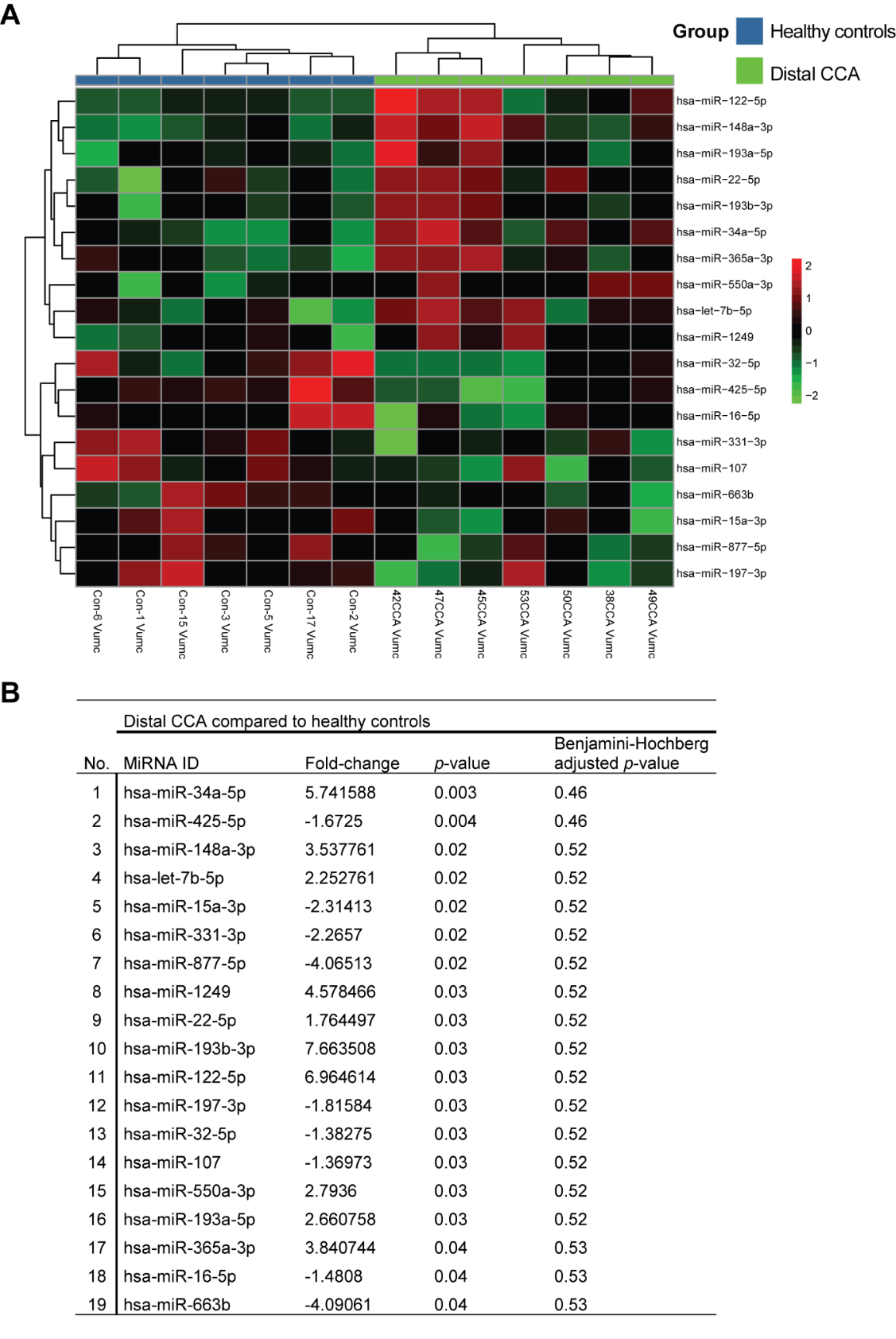


**Figure 1. A.** Heat map diagram showing one-way hierarchical clustering of the differently expressed miRNAs (*P* < 0.05) emerging from the microarray analysis. The heat map reports relative levels of miRNA expression in a green (lower expression) to red (higher expression) scale across all samples. **B.** Overview of the significantly differently expressed miRNAs in plasma samples of patients with distal CCA compared to healthy controls. Distal CCA = distal cholangicarcinoma, miRNA = microRNA, hsa-miR = human microRNA.


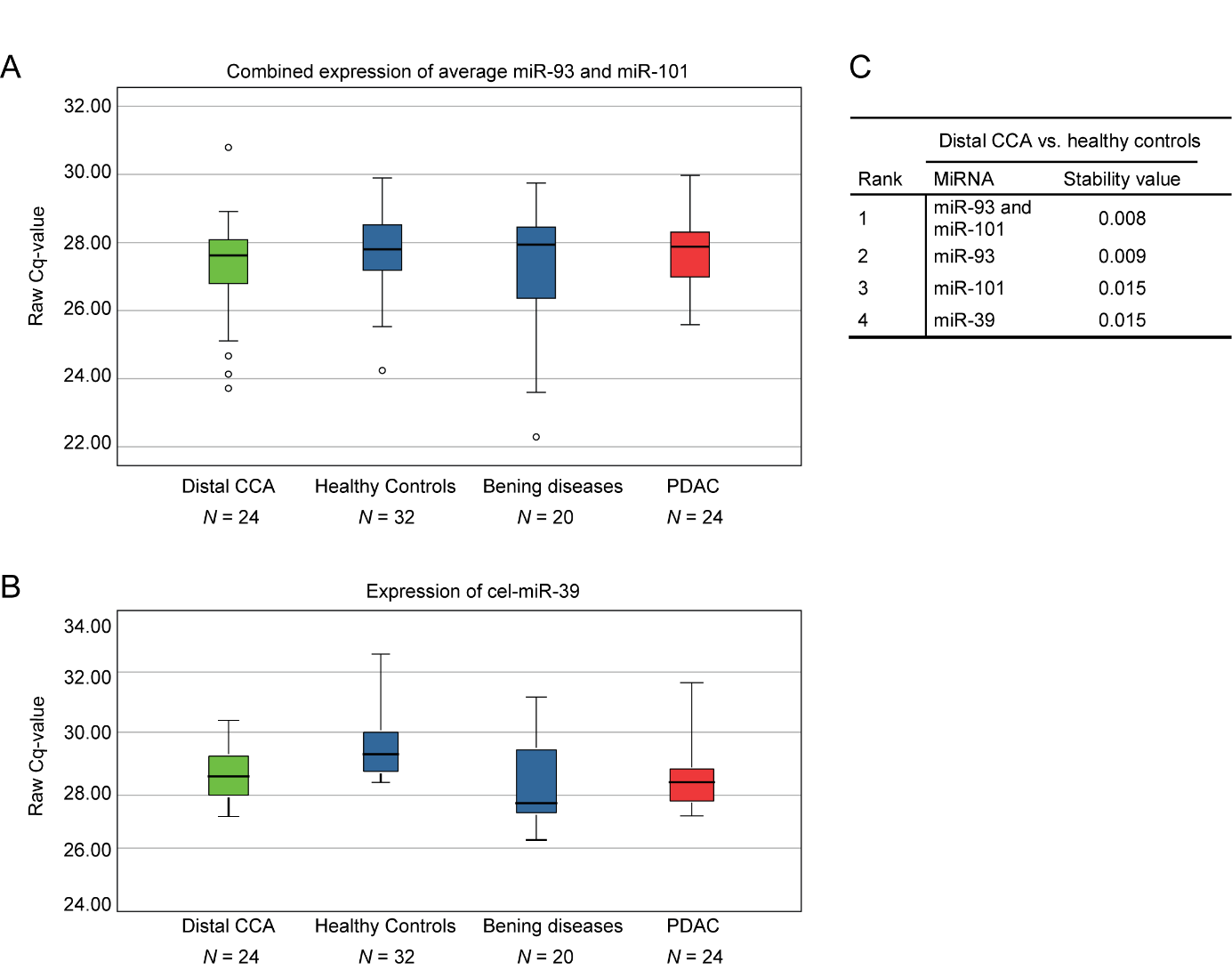


**Figure 2.** MiRNA expression of the reference gene combination miR-93 and miR-101 (**A**) and cel-miR-39 (**B**) in the evaluation phase and validation phase. Box plots are displayed for the average raw Cq-values, with the horizontal lines representing the mean ± SD. The table (**C**) displays the stability values of the potential reference miRNAs, as calculated by NormFinder.


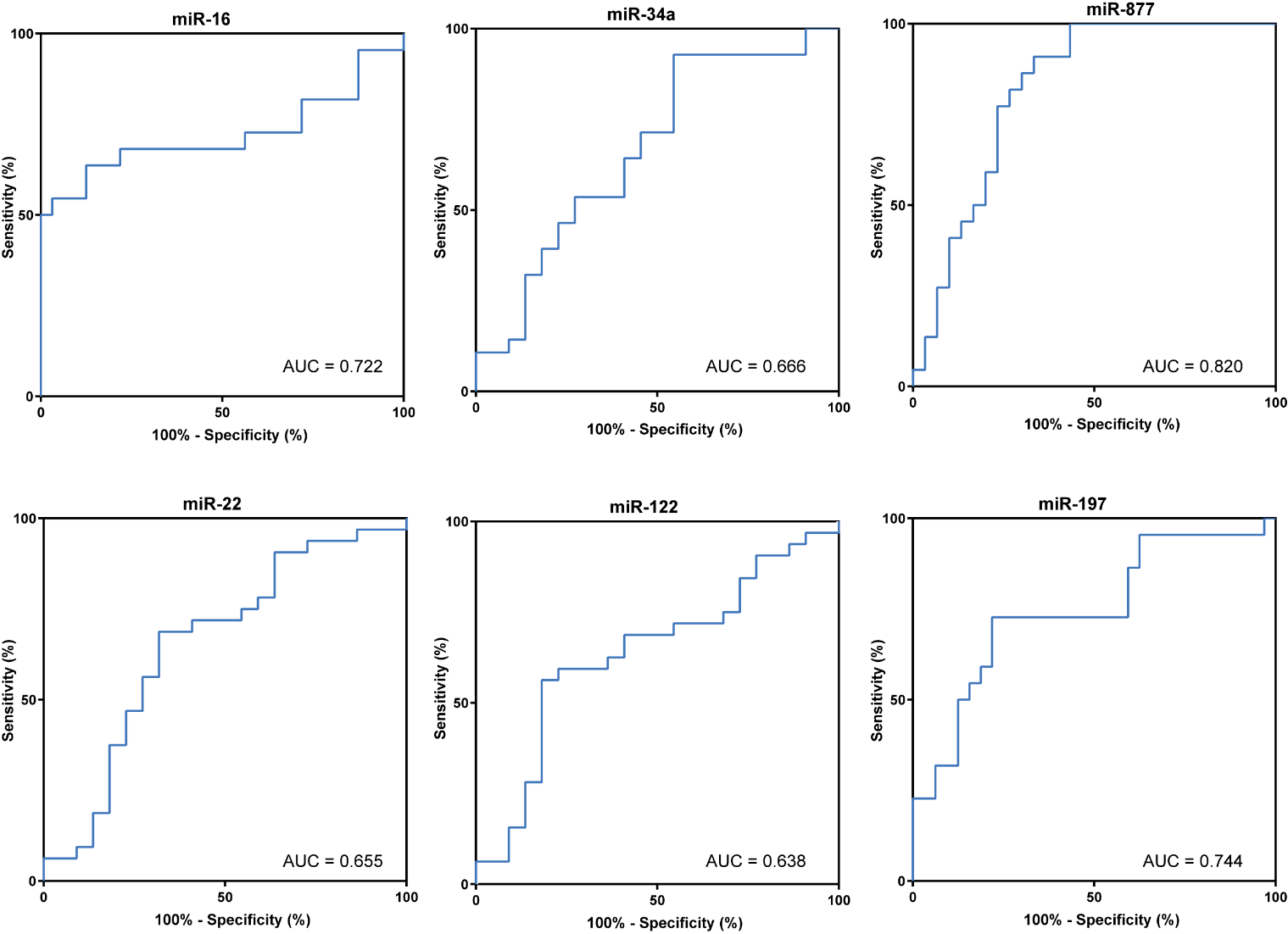


**Figure 3.** ROC-curves of the individual microRNAs evaluated in the evaluation phase, comparing distal CCA to healthy controls. ROC-curves with AUC are displayed.

**
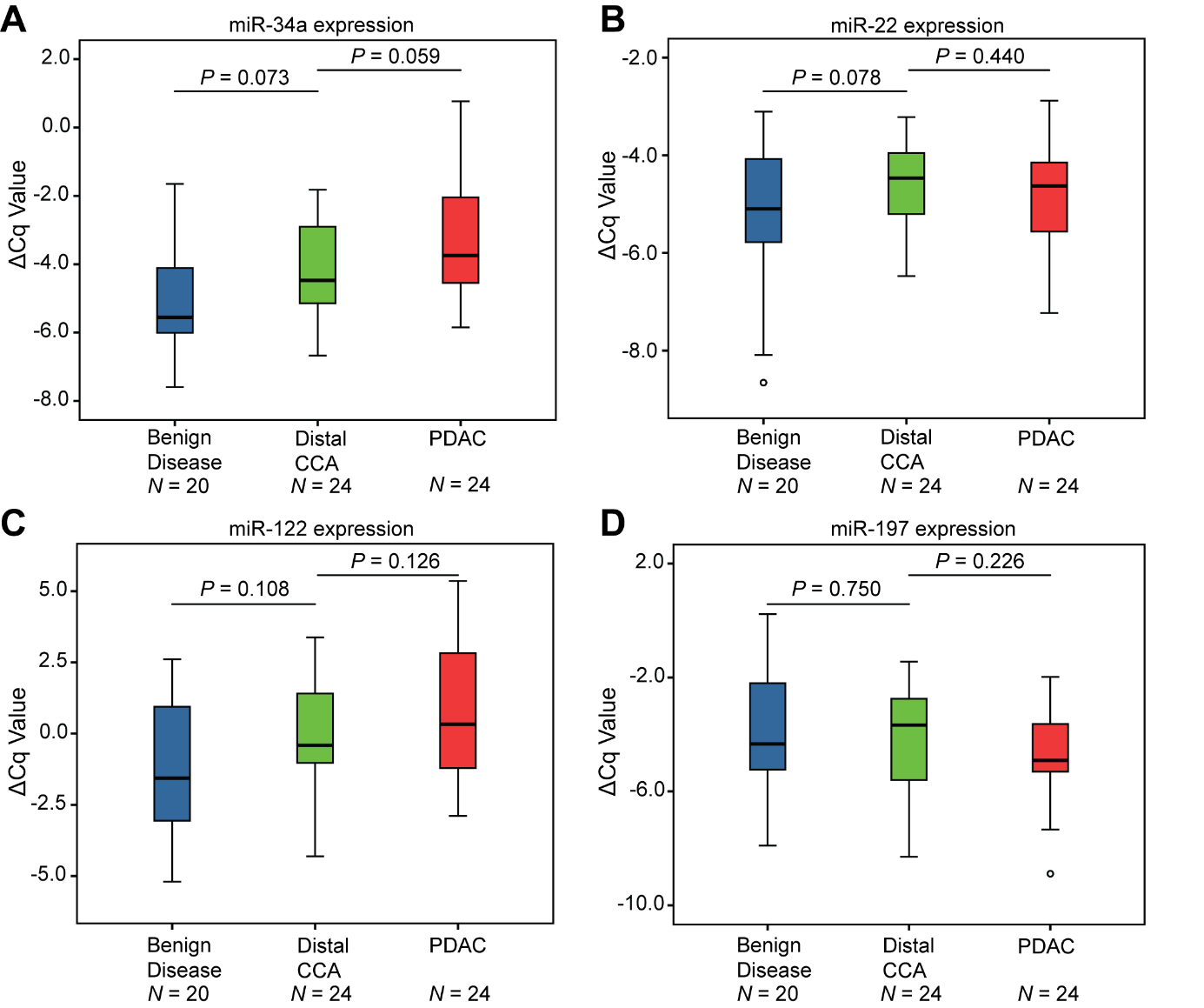
 Figure 4.** Individual miRNA expression profiles of the miRNAs investigated in the validation phase.Expression levels of miR-34a (**A**), miR-22 (**B**), miR-122 (**C**), and miR-197 (**D**) comparing distal CCA to benign disease and PDAC. No significant differences were found for these miRNAs.Normalized Cq (ΔCq) are shown, box plots are displayed for the average ΔCq-values, with the horizontal lines representing the mean ± SD. Distal CCA = distal cholangiocarcinoma, PDAC = Pancreatic Ductal Adenocarcinoma.


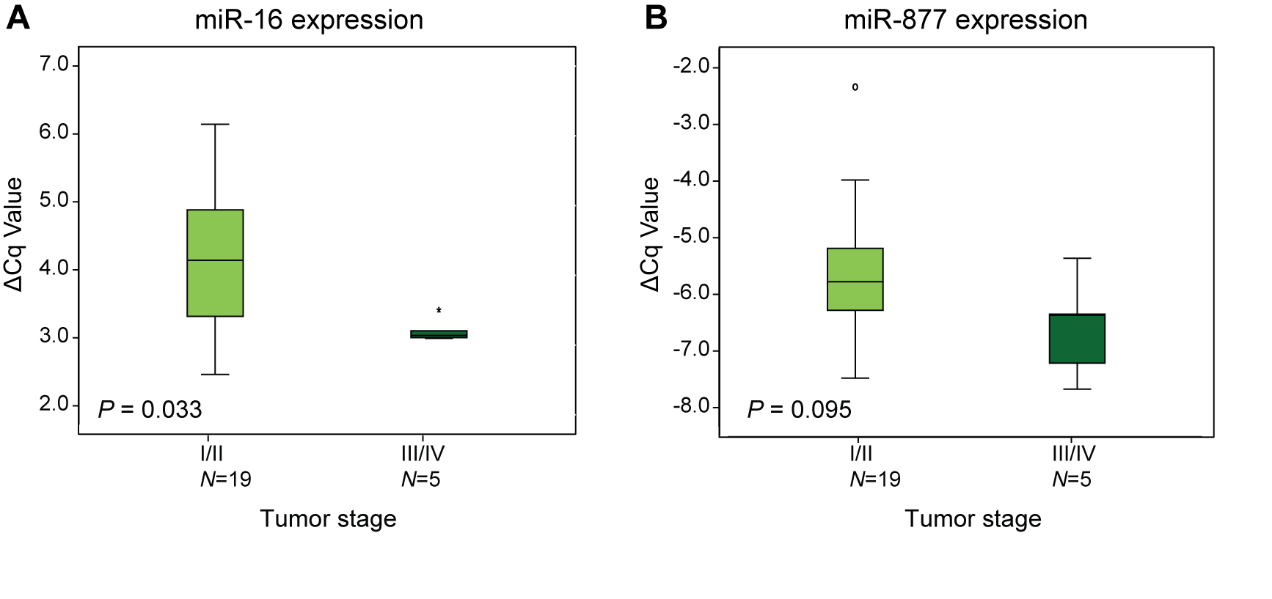


**Figure 5.** Expression of miR-16 (**A**) and miR-877 (**B**) in early-stage (I/II) and late-stage (III/IV) distal cholangiocarcinoma. Expressions of miR-16 was significantly lower in late-stage distal cholangiocarcinoma.

**
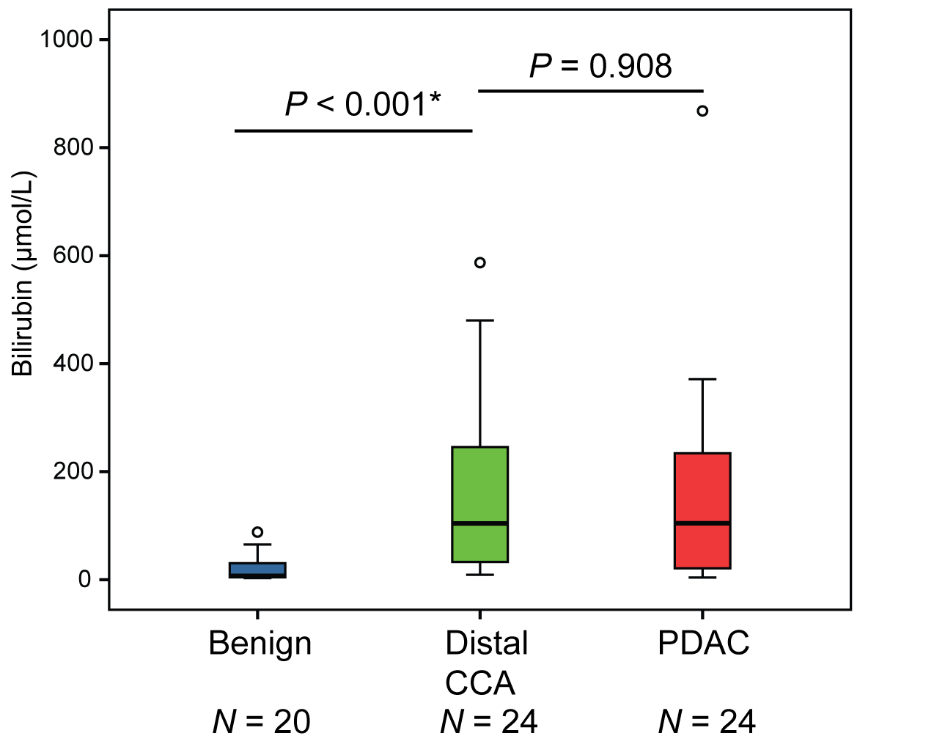
.**

**Figure 6.** Expression of bilirubin levels in patients with benign disease versus distal CCA and distal CCA versus PDAC. Distal CCA = distal cholangiocarcinoma, PDAC = pancreatic ductal adenocarcinoma. * indicates significant *P*-value.


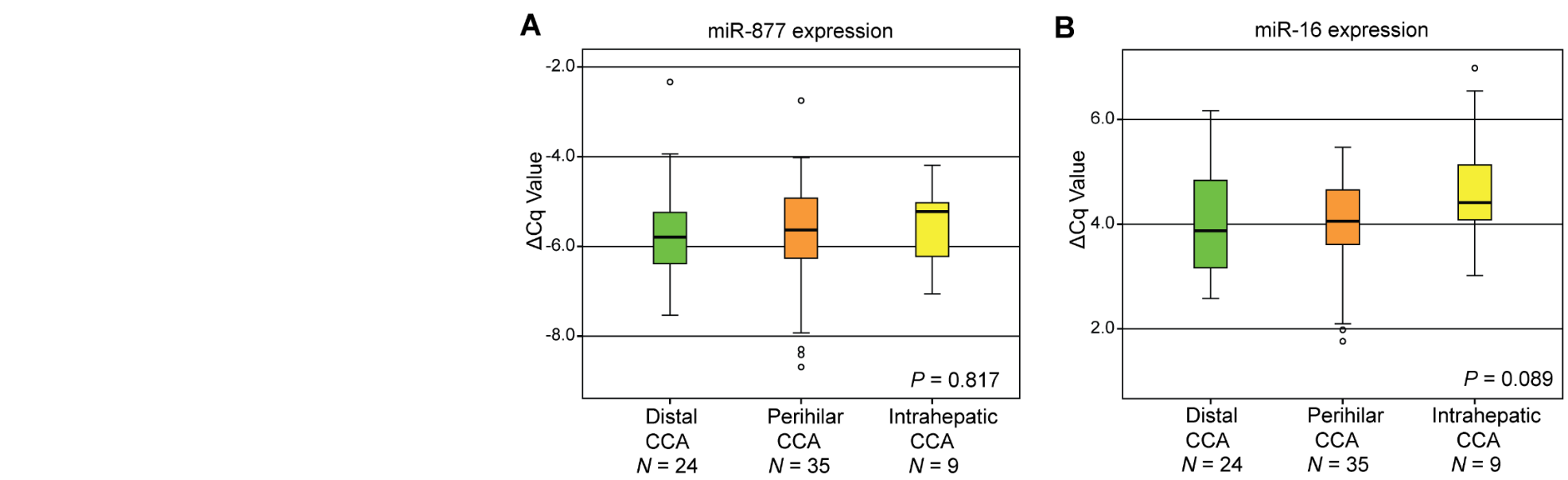
.

**Figure 7.** Expression levels of miR-877 (**A**) and miR-16 (**B**) in plasma samples of patients with distal CCA compared to perihilar and intrahepatic CCA. No significant differences were found between the expression levels of the groups. Normalized Cq (ΔCq) are shown, box plots are displayed for the average Cq-values, with the horizontal lines representing the mean ± SD. CCA = cholangiocarcinoma.

**Table 1.** Overview of the 12 miRNAs selected for evaluation and the *P*-value comparing distal CCA to healthy controls in the evaluation phase.

| **miRNA** | ***P*-value** |
| --- | --- |
| miR-16-5p | 0.021 |
| miR-15a-3p | NS |
| miR-21-5p | NS |
| miR-22-5p | 0.068 |
| miR-32-5p | NS |
| miR-34a-5p | 0.004 |
| miR-122-5p | 0.048 |
| miR-148-5p | NS |
| miR-150-5p | NS |
| miR-197-3p | 0.001 |
| miR-331-3p | NS |
| miR-877-5p | <0.001 |

**Table 2.** Predicted gene targets of miR-16-5p and miR-877-5p.

| **MiRNA_ID** | **Refseq_id** | **Gene_symbol** | **start** | **end** | **n_pairings** | **position** | **Validated** |
| --- | --- | --- | --- | --- | --- | --- | --- |
| hsa-miR-16-5p | NM_001667 | ARL2 | 764 | 779 | 14 | 3UTR | MIRT001478 |
| hsa-miR-16-5p | NM_004056 | CA8 | 1389 | 1402 | 12 | 3UTR | MIRT559009 |
| hsa-miR-16-5p | NM_001759 | CCND2 | 5831 | 5854 | 19 | 3UTR | MIRT003431 |
| hsa-miR-16-5p | NM_020240 | CDC42SE2 | 1372 | 1397 | 16 | 3UTR | MIRT256306 |
| hsa-miR-16-5p | NM_001177381 | CPEB2 | 3607 | 3629 | 18 | 3UTR | MIRT164261 |
| hsa-miR-16-5p | NM_001278542 | DIXDC1 | 1248 | 1285 | 20 | 3UTR | MIRT051092 |
| hsa-miR-16-5p | NM_001968 | EIF4E | 10588 | 10610 | 15 | 3UTR | MIRT001469 |
| hsa-miR-16-5p | NM_001349798 | FBXW7 | 4078 | 4101 | 19 | 3UTR | MIRT032006 |
| hsa-miR-16-5p | NM_017423 | GALNT7 | 2666 | 2720 | 13 | 3UTR | MIRT001466 |
| hsa-miR-16-5p | NM_004798 | KIF3B | 5487 | 5502 | 13 | 3UTR | MIRT152737 |
| hsa-miR-16-5p | NM_203403 | LURAP1L | 1756 | 1772 | 15 | 3UTR | MIRT556382 |
| hsa-miR-16-5p | NM_001270384 | MIGA1 | 2206 | 2249 | 17 | 3UTR | MIRT727699 |
| hsa-miR-16-5p | NM_001184748 | PAFAH1B2 | 1706 | 1722 | 12 | 3UTR | MIRT001434 |
| hsa-miR-16-5p | XM_005262769 | PAQR3 | 1818 | 1842 | 16 | 3UTR | MIRT032065 |
| hsa-miR-16-5p | NM_014338 | PISD | 1567 | 1585 | 12 | 3UTR | MIRT001431 |
| hsa-miR-16-5p | NM_001330167 | RAB11FIP2 | 5794 | 5807 | 11 | 3UTR | MIRT726823 |
| hsa-miR-16-5p | NM_016370 | RAB9B | 1141 | 1171 | 19 | 3UTR | MIRT000803 |
| hsa-miR-16-5p | NM_003194 | TBP | 1525 | 1536 | 10 | 3UTR | MIRT051286 |
| hsa-miR-16-5p | NM_001136554 | TLK1 | 4219 | 4263 | 17 | 3UTR | MIRT031590 |
| hsa-miR-16-5p | XM_017005934 | TMCC1 | 3401 | 3425 | 20 | 3UTR | MIRT031658 |
| hsa-miR-16-5p | NM_019116 | UBFD1 | 1782 | 1793 | 10 | 3UTR | MIRT032064 |
| hsa-miR-16-5p | NM_001319238 | ZC3H11A | 5194 | 5215 | 18 | 3UTR | MIRT031561 |
| hsa-miR-877-5p | NM_004985 | KRAS | 3505 | 3534 | 19 | 3UTR | MIRT502087 |
| hsa-miR-877-5p | NM_005775 | SORBS3 | 2669 | 2692 | 18 | 3UTR | MIRT037354 |

**Table 3.** Clinicopathological characteristics of the included patients with distal CCA, perihilar CCA, and intrahepatic CCA.

|  | Distal  CCA  (*N* = 24) | Perihilar  CCA  (*N* = 35) | Intrahepatic  CCA  (*N* = 9) | *P*-value* |
| --- | --- | --- | --- | --- |
| **Age – yrs** |  |  |  | 0.298 |
| Mean (± SD) | 68 (± 11) | 66 (± 9) | 62 (± 7) |  |
| **Sex – No. (%)** |  |  |  | 0.028* |
| Male | 15 (63) | 11 (31) | 6 (67) |  |
| Female | 9 (37) | 24 (69) | 3 (33) |  |
| **Stage^+^ – No. (%)** |  |  |  | 0.002* |
| I | 2 (8) | 1 (3) | 0 (0) |  |
| II | 17 (71) | 10 (29) | 2 (22) |  |
| III | 2 (8) | 19 (54) | 1 (11) |  |
| IV | 3 (13) | 5 (14) | 3 (33) |  |
| Missing | 0 (0) | 0 (0) | 3 (33) |  |
| **CA19-9 – No. (%)** |  |  |  | 0.318 |
| Normal § | 6 (25) | 7 (20) | 1 (11) |  |
| ULN to <59 × ULN | 15 (63) | 20 (57) | 5 (56) |  |
| High ≥59 × ULN | 1 (4) | 5 (14) | 3 (33) |  |
| Missing | 2 (8) | 3 (9) | 0 (0) |  |
| **CA19-9 (U/mL)** |  |  |  | 0.456 |
| Median  (± SD) | 86  (± 844) | 237  (± 3,900) | 217  (± 35,498) |  |
| **Bilirubin – No. (%)** |  |  |  | 0.005* |
| High | 21 (87) | 27 (77) | 3 (33) |  |
| Low | 3 (13) | 8 (23) | 6 (67) |  |
| Missing | 0 (0) | 0 (0) | 0 (0) |  |
| **Bilirubin (µmol/L)** |  |  |  | 0.746 |
| Median  (± SD) | 104  (± 166) | 103  (± 134) | 12  (± 153) |  |
| *Indicates significant *P­*-value  ^+^AJCC Cancer Staging Manual, 7^th^ Edition  CCA = cholangiocarcinoma, CA19-9 = carbohydrate antigen 19-9,  ULN = Upper Limit of Normal, §The normal range was 0 - 37 U per milliliter,  No. = number of patients. | | | | |
