# Supplementary material for: Unravelling the Diagnostic Dilemma: A MicroRNA Panel of Circulating MiR-16 and MiR-877 as A Diagnostic Classifier for Distal Bile Duct Tumors
Source: Cancers (Basel). 2019 Aug 15;11(8):1181. doi: 10.3390/cancers11081181 (PMC6721566; doi:10.3390/cancers11081181)
Supplement: Supplementary file 1 [file cancers-11-01181-s001.zip › cancers-563234-supplyment update/cancers-563234-Supply Methods and Product Information--.docx]

Appendix A—Supplementary methods

Sample collection

Between 2014–2018, blood was collected prospectively at the Cancer Center Amsterdam of the Amsterdam UMC, VU University Amsterdam (NL) and University of Amsterdam (NL) from all consecutive patients presenting with suspected pancreatic and hepatobiliary lesions. After radiological imaging and careful histopathological confirmation of malignant lesions, patients with distal CCA, perihilar CCA, intrahepatic CCA, PDAC, and BD were included for further analysis. Tumor tissues of all included patients with distal CCA and PDAC were available for histopathological confirmation and before study inclusion, all samples were revised by blinded pathologists (NF/NG). In unresectable cases, tissue samples of either the primary tumor were obtained by staging laparoscopy (open–close procedures in locally advanced or metastatic cases) or tissue biopsies of the metastatic lesion (for example, liver metastases). Also, if histopathology was inconclusive, an additional staining of Cytokeratin-17 (KRT17), Annexin A10 (ANXA10) and parathymosin (PTMS), would be considered, using the KRT17+/ANXA10+/PTMS- staining pattern as a biomarker panel for the correct diagnosis of PDAC and KRT17−/ANXA10−/PTMS+ for the diagnosis of CCA, as described recently [1].

BD included patients with chronic pancreatitis (N = 9), choledocholithiasis (N = 6), and cholangitis (N = 5). Of the patients with chronic pancreatitis, 8 out of 9 patients underwent surgical resection and histopathological confirmation. For choledocholithiasis and cholangitis, a biliary stricture was present on imaging and these patients were suspected for malignancy, but were confirmed as having benign lesions by cytology and follow-up or resection of the suspected lesion. All patients with cholangitis underwent resection of the biliary lesion due to suspicion of malignancy. Of the patients with cholangitis, two patients demonstrated IgG4-mediated cholangitis and three patients sclerosing cholangitis. Patients with premalignant lesions or any other existent malignancies were omitted from final inclusion. Healthy individuals were enrolled as controls, provided that they showed no clinical evidence of hepatopancreaticobiliary disease and had no known history of any type of malignancy.

Sample collection, plasma RNA extraction

Patient blood was drawn at diagnosis into sterile 6 mL EDTA-coated tubes (BD Vacutainer CPT). Whole blood was first centrifuged for 20 min at 120 × g. Next, platelet-depleted plasma was centrifuged for 10 min at 5000 × g. Aliquots were stored at −80 ºC in a prospectively maintained biobank for long-term storage. Plasma samples with visual signs of hemolysis or higher absorbance at 414 nm measured by spectral analyses were excluded from further investigation. Total RNA was extracted from 300 µL of plasma using the miRCURY RNA Isolation Kit-Biofluids (Exiqon, Vedbaek, Denmark) according to the manufacturer’s protocol, which included DNase treatment. For RNA isolation of samples included in the evaluation and validation phases, 1 µg of MS2 bacteriophage carrier RNA (Roche, Castle Hill, NSW, Australia) and 20 fmol cel-miR-39-3p synthetic RNA Spike-In template (Exiqon, Vedbaek, Denmark) were added to the lysis solution (final volume 60µL). Cel-miR-39 was added as an internal control to assess RNA isolation efficiency with respect to technical variation. Using a NanoDrop 1000 spectrophotometer, RNA yield was quantified and the purity evaluated at the 260/280 nm ratio. RNA was stored at −80 ºC until further processing.

MiRNA profile screening in the discovery phase by PCR panel analysis

MiRNA profiling and RNA quality controls of the discovery phase samples were conducted by Exiqon Services, Denmark. From each sample, RNA was reverse-transcribed to complementary DNA (cDNA). cDNA was diluted 50 times and assayed in 10 µL PCR reactions using the miRCURY LNA Universal RT microRNA PCR, Polyadenylation and cDNA synthesis kit (Exiqon, Vedbaek, Denmark) according the manufacturer’s protocol. Using the ExiLENT SYBR Green Master Mix (Exiqon, Vedbaek, Denmark), 752 miRNAs were assayed on the miRNA Ready-to-Use PCR, Human Panel I+II, V4 (Exiqon, Vedbaek, Denmark) by RT-qPCR. RT-qPCR was run in a LightCycler 480 Real-Time PCR System (Roche). Data quality control included assessment of hemolysis by the ratio of two miRNAs: miR-451 (expressed by red blood cells) and miR-23a (stable in samples not influenced by hemolysis). Samples with high ratios (>7) were excluded. The amplification curves were analyzed using the Roche LC software for determination of the Cq value and for melting curve analysis. The amplification efficiency was calculated using algorithms similar to LinReg software. Assays with quantification cycles (Cqs) higher than 37 or with a difference of less than 5 Cqs with negative controls were excluded from further analysis. To reduce technical bias, raw data were normalized to the global mean by subtracting the average of assays detected in all samples from the sample assay Cq (∆Cq) [2]. Following normalization, differential miRNA expression between distal CCA and healthy controls was calculated using the ∆∆Cq method [3]. The panel data have been uploaded to the GEO database (accession number GSE117687).

Validation of miRNAs in the evaluation and validation phase by RT-qPCR

To validate candidate miRNAs identified by PCR panel profiling, an independent cohort of patients was used. RNA samples were reverse-transcribed to cDNA using the Universal cDNA Synthesis Kit II (Exiqon, Vedbaek, Denmark) in accordance with the manufacturer’s protocol. Next, RT-qPCR was performed using ExiLENT SYBR Green Master Mix (Exiqon, Vedbaek, Denmark) and LNA-enhanced primers (Exiqon, Vedbaek, Denmark) on the CFX Manager CFX96 (Bio-Rad), in line with the accompanying protocol (Supplementary Product Information). Cqs were automatically calculated with CFS Manager Software (Bio-Rad). Technical duplicates were performed for all miRNAs.

Based on previous reports regarding identification of suitable reference genes, a set of four candidate normalizing reference genes was selected for normalization of raw output data in the validation phase, including miR-93, miR-101, miR-39 and miR-1228 [4–8]. In order to find the most stable (combination of) reference gene(s), NormFinder was used to assess the expression stability of potential reference miRNAs [9]. MiRNA expression was normalized by subtracting the average Cq value of the miRNA of interest from the average Cq value of the emerging reference genes, to obtain the ∆Cq. Fold change was expressed as 2∆∆Cq and − 2-∆∆Cq for positive and negative ∆Cq, respectively.

Appendix B—Supplementary Product Information

| Company | Country | Product number | Product name |
| --- | --- | --- | --- |
| Exiqon | Denmark | 203301 | Universal cDNA Synthesis Kit II, 8-64 rxns |
| Exiqon | Denmark | 203421 | ExiLENT SYBR® Green master mix, 20ml |
| Exiqon | Denmark | 203952 | cel-miR-39-3p, LNA™ control primer set, UniRT |
| Exiqon | Denmark | 204230 | hsa-miR-21-5p LNA™ PCR primer set, UniRT |
| Exiqon | Denmark | 204255 | hsa-miR-22-5p LNA™ PCR primer set, UniRT |
| Exiqon | Denmark | 204380 | hsa-miR-197-3p LNA™ PCR primer set, UniRT |
| Exiqon | Denmark | 204435 | hsa-miR-15a-3p LNA™ PCR primer set, UniRT |
| Exiqon | Denmark | 204486 | hsa-miR-34a-5p LNA™ PCR primer set, UniRT |
| Exiqon | Denmark | 204660 | hsa-miR-150-5p LNA™ PCR primer set, UniRT |
| Exiqon | Denmark | 204715 | hsa-miR-93-5p LNA™ PCR primer set, UniRT |
| Exiqon | Denmark | 204786 | hsa-miR-101-3p LNA™ PCR primer set, UniRT |
| Exiqon | Denmark | 204792 | hsa-miR-32-5p LNA™ PCR primer set, UniRT |
| Exiqon | Denmark | 205626 | hsa-miR-877-5p LNA™ PCR primer set, UniRT |
| Exiqon | Denmark | 205664 | hsa-miR-122-5p LNA™ PCR primer set, UniRT |
| Exiqon | Denmark | 205702 | hsa-miR-16-5p LNA™ PCR primer set, UniRT |
| Exiqon | Denmark | 205867 | hsa-miR-148a-3p LNA™ PCR primer set, UniRT |
| Exiqon | Denmark | 206046 | hsa-miR-331-3p LNA™ PCR primer set, UniRT |
| Exiqon | Denmark | 300112 | miRCURY RNA Isolation Kit - Biofluids (50) |
| Exiqon | Denmark | 2111995 | hsa-miR-1228-3p LNA™ PCR primer set, UniRT |
| Bio-Rad | USA | HSP9645 | Hard-shell 96-well PCR plates, white well, green shell GHX |
| Bio-Rad | USA | MSB1001 | Microseal® 'B' PCR Plate Sealing Film, adhesive |

References

1. Takenami, T.; Maeda, S.; Karasawa, H.; Suzuki, T.; Furukawa, T.; Morikawa, T.; Takadate, T.; Hayashi, H.; Nakagawa, K.; Motoi, F.; et al. Novel biomarkers distinguishing pancreatic head Cancer from distal cholangiocarcinoma based on proteomic analysis. *BMC* *Cancer* **2019**, *19*, 318.
2. Mestdagh, P.; Van Vlierberghe, P.; De Weer, A.; Muth, D.; Westermann, F.; Speleman, F.; Vandesompele, J. A novel and universal method for microRNA RT-qPCR data normalization. *Genome* *Biol.* **2009**, *10*, R64.
3. Livak, K.J.; Schmittgen, T.D. Analysis of relative gene expression data using real-time quantitative PCR and the 2 (-Delta Delta C(T)) Method. *Methods* **2001**, *25*, 402–408.
4. Pei, Z.; Liu, S.-M.; Huang, J.-T.; Zhang, X.; Yan, D.; Xia, Q.; Ji, C.; Chen, W.; Zhang, X.; Xu, J.; et al. Clinically relevant circulating microRNA profiling studies in pancreatic cancer using meta-analysis. *Oncotarget* **2017**, *8*, 22616–22624.
5. Schwarzenbach, H.; Da Silva, A.M.; Calin, G.; Pantel, K. Data Normalization Strategies for MicroRNA Quantification. *Clin.* *Chem.* **2015**, *61*, 1333–1342.
6. Hu, J.; Wang, Z.; Liao, B.Y.; Yu, L.; Gao, X.; Lu, S.; Wang, S.; Dai, Z.; Zhang, X.; Chen, Q.; et al. Human miR-1228 as a stable endogenous control for the quantification of circulating microRNAs in cancer patients. *Int.* *J.* *Cancer* **2014**, *135*, 1187–1194.
7. Niu, Y.; Wu, Y.; Huang, J.; Li, W.; Kang, K.; Qu, J.; Gou, D. Identification of reference genes for circulating microRNA analysis in colorectal cancer. *Sci.* *Rep.* **2016**, *6*, 35611.
8. Song, J.; Bai, Z.; Han, W.; Zhang, J.; Meng, H.; Bi, J.; Ma, X.; Han, S.; Zhang, Z. Identification of suitable reference genes for qPCR analysis of serum microRNA in gastric cancer patients. *Dig.* *Dis.* *Sci.* **2012**, *57*, 897–904.
9. Andersen, C.L.; Jensen, J.L.; Orntoft, T.F. Normalization of real-time quantitative reverse transcription-PCR data: A model-based variance estimation approach to identify genes suited for normalization, applied to bladder and colon cancer data sets. *Cancer* *Res.* **2004**, *64*, 5245–5250.
