# Supplementary figures and images for: Unravelling the Diagnostic Dilemma: A MicroRNA Panel of Circulating MiR-16 and MiR-877 as A Diagnostic Classifier for Distal Bile Duct Tumors
Source: Cancers (Basel). 2019 Aug 15;11(8):1181. doi: 10.3390/cancers11081181 (PMC6721566; doi:10.3390/cancers11081181)

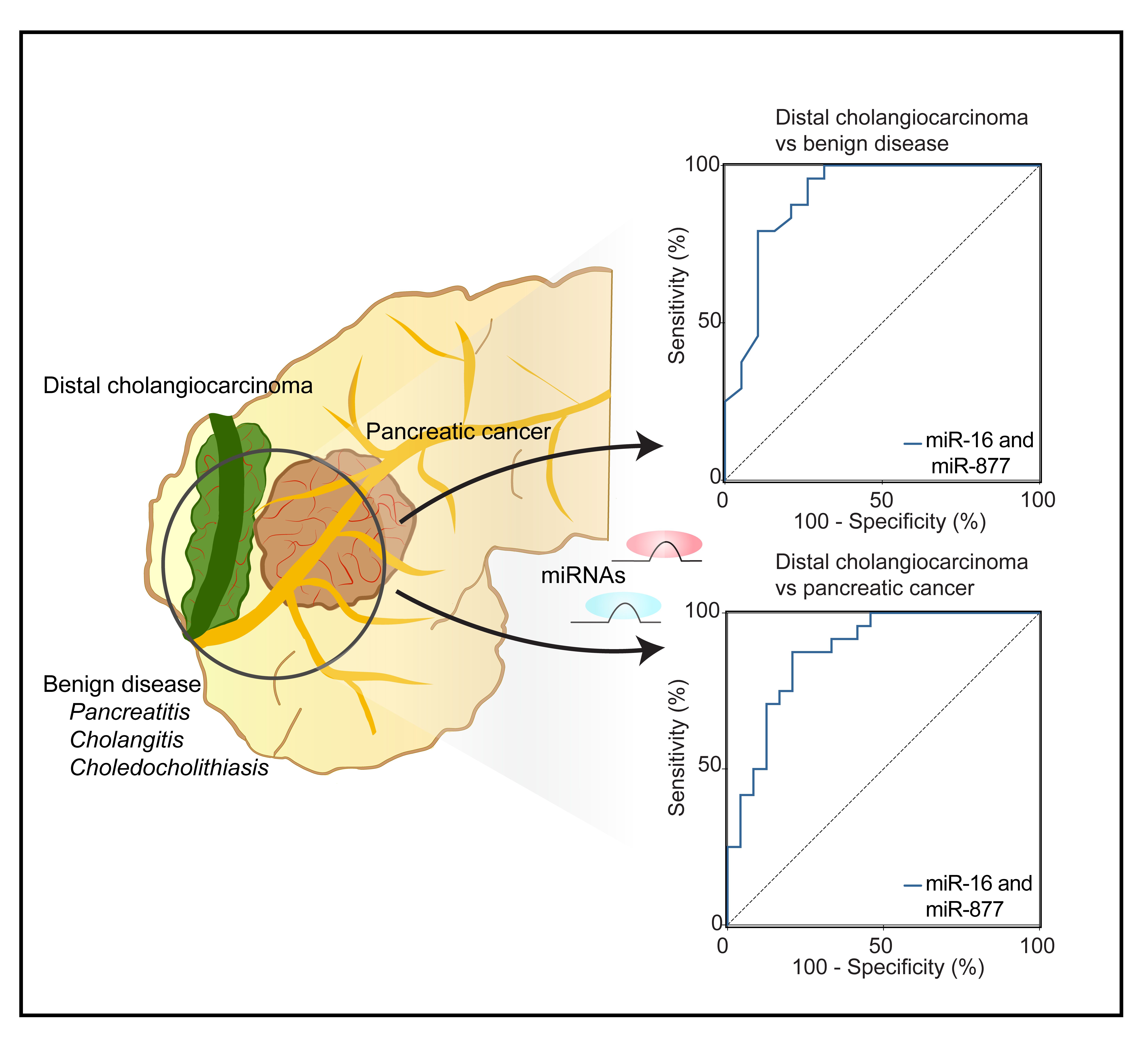

Supplement: Supplementary file 1 [file cancers-11-01181-s001.zip › cancers-563234-supplyment update/cancers-563234-GA.jpg]
